# iTAG an optimized IMiD-induced degron for targeted protein degradation in human and murine cells

**DOI:** 10.1016/j.isci.2023.107059

**Published:** 2023-06-07

**Authors:** Habib Bouguenina, Stephanos Nicolaou, Yann-Vaï Le Bihan, Elizabeth A. Bowling, Cheyenne Calderon, John J. Caldwell, Brinley Harrington, Angela Hayes, P. Craig McAndrew, Costas Mitsopoulos, Fernando Jr. Sialana, Andrea Scarpino, Mark Stubbs, Arjun Thapaliya, Siddhartha Tyagi, Hannah Z. Wang, Francesca Wood, Rosemary Burke, Florence Raynaud, Jyoti Choudhary, Rob L.M. van Montfort, Amine Sadok, Thomas F. Westbrook, Ian Collins, Rajesh Chopra

**Affiliations:** 1 Centre for Cancer Drug Discovery, the Institute of Cancer Research, 15 Cotswold Road, Sutton, London SM2 5NG, UK; 2 Therapeutic Innovation Centre (THINC), Verna and Marrs McLean Department of Biochemistry and Molecular Biology, Baylor College of Medicine, Houston, TX 77030, USA; 3 Functional Proteomics Group, The Institute of Cancer Research, Chester Beatty Laboratories, London SW3 6JB, UK

**Keywords:** Oncology, Biological sciences, Biochemistry, Cell biology

## Abstract

To address the limitation associated with degron based systems, we have developed iTAG, a synthetic tag based on IMiDs/CELMoDs mechanism of action that improves and addresses the limitations of both PROTAC and previous IMiDs/CeLMoDs based tags. Using structural and sequence analysis, we systematically explored native and chimeric degron containing domains (DCDs) and evaluated their ability to induce degradation. We identified the optimal chimeric iTAG(DCD23 60aa) that elicits robust degradation of targets across cell types and subcellular localizations without exhibiting the well documented “hook effect” of PROTAC-based systems. We showed that iTAG can also induce target degradation by murine CRBN and enabled the exploration of natural neo-substrates that can be degraded by murine CRBN. Hence, the iTAG system constitutes a versatile tool to degrade targets across the human and murine proteome.

## Highlights

• IMiD-induced degron sequences from various neo-substrates were evaluated
• ITAG a 60 amino acids tag derived from Ikaros and ZFP91 was selected as optimal
• ITAG induces the degradation of various targets in human and murine cells
• Thalidomide derivatives can induce target degradation in mouse cells (FPFT2216 PDE6D)

## Graphical abstract

**Figure:**
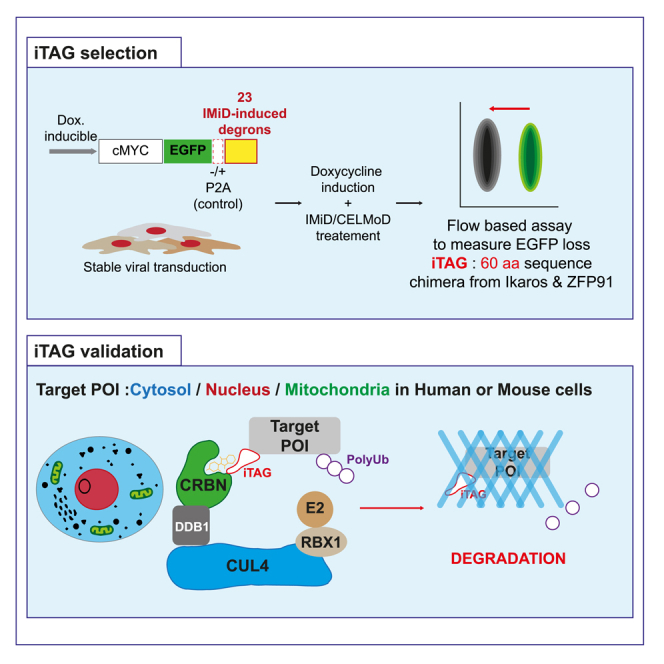

## Introduction

The study of protein function in human health and disease is being transformed by the use of chemical biology and protein degradation systems.^1^^,^^2^ Current methods for evaluating protein function employ either small molecule chemical probes or genetic silencing, which are powerful tools, but also present limitations. Small molecule probes allow for time- and dose-dependent control over target modulation enabling a pharmacologically relevant assessment of protein function. In addition, removal of the chemical probe through washout experiments enables the recovery of target function after modulation which is necessary for establishing causal relationships.^3^ However, small molecule inhibitors and agonists are currently restricted to a few targets (estimated to be 4% of the proteome),^4^ often lack specificity and generality by targeting the catalytic activity of proteins but failing to inhibit non-catalytic functions. On the other hand, genetic methods for modulating protein expression levels, such as RNA interference and CRISPR/Cas9-genome editing, have been extensively used for target validation, with available libraries targeting every gene and splice variant in multiple species.^5^^,^^6^^,^^7^ The protocols for these approaches are constantly being improved enabling a cost-effective approach to studying target function. Genetic disruption of the targets is, however, associated with variability in knockdown efficiency, off-target effects and, in the case of CRISPR/Cas9-genome editing, require clonal selection which makes it hard to distinguish between phenotypes directly related to the loss of target protein and those related to cellular adaptation as a consequence of loss of function. Therefore to attribute a causal relationship, the phenotypic consequence must be then reversed or rescued by target cDNA re-expression through gene transfer experiments.^3^ Furthermore, the use of genetic methods to knock down targets *in vivo* in a cell specific and inducible manner remains a challenging process.

Recently, chemical biology tools have been developed to modulate protein levels directly and acutely by affecting their stability in cells. These technologies are based on the use of small amino acid tags that are fused to a protein of interest and that will induce its degradation in the presence of a specific chemical compound. Examples include: small-molecule displacement of a cryptic degron,^8^ Small Molecule-Assisted Shutoff (SMASh),^9^ degradation of a fused hydrophobic tag (HaloTag),^10^ the auxin-inducible degron (AID),^11^^,^^12^ the HaloPROTAC^13^^,^^14^ and dTAG system.^15^^,^^16^ These tools have enabled acute protein degradation and demonstration of a relationship between loss of a protein of interest and early cellular changes.^1^ The broad adoption of these tools has been limited, due to multiple factors that include: the multi-component complexity of some systems, such as the AID that requires the expression of both the AID tag and the plant TIR E3 ligase; low versatility, such as SMASh system, which is limited to proteins with short half-lives. Limits of the PROTAC-based tools (HaloPROTAC, dTAG) relate to the need to use high molecular weight PROTACs with significant compound cost, restricted dose-response modulation because of the “hook effect” and limited characterization of *in vivo* pharmacokinetic (PK) and pharmacodynamic (PD) properties. Alternative systems, such as Trim-Away,^17^ have enabled the degradation of endogenous targets using antibodies recognized by the E3 TRIM21 but require the preparation and delivery of target specific antibodies in cells.

One of the best described examples of drug-induced protein degradation is the mechanism of action of the thalidomide-like derivatives (called immunomodulatory drugs IMiDs, or more recently, Cereblon E3 ligase modulators CELMoDs). These degrader drugs bind to a conserved tri-tryptophan cage on the surface of Cereblon (CRBN), the substrate receptor of the Cullin4-RING E3 ubiquitin ligase CRL4^CRBN^, and induce the alteration of CRL4^CRBN^ substrate specificity, leading to the recruitment, ubiquitination and subsequent proteasomal degradation of neo-substrates such as Ikaros (IKZF1), Aiolos (IKZF3), Casein Kinase 1 alpha (CK1α or CSNK1A), G_1_ to S phase transition protein 1 (GSPT1) and zinc finger protein 91 (ZFP91)^18^^,^^19^^,^^20^^,^^21^^,^^22^^,^^23^^,^^24^ (Table S1). Neo-substrates interact with the degrader-bound surface of CRBN through recognition motifs known as degrons, characterized by a common β-hairpin loop with a conserved glycine at the apex, which is crucial for the interaction of the degron with the degrader compound. The amino acid sequences adjacent to the sentinel glycine in the β-hairpin loop are variable among neo-substrates and are critical for the specificity of recruitment and degradation of neo-substrates by a particular CRBN binding agent (Table S1). In the case of the neo-substrates Ikaros, Aiolos and ZFP91, the degron motif is located within a C2H2 zinc finger (ZF) domain, with the first half of the domain harboring a CxxCG sequence signature containing the conserved glycine just after the second Cysteine of the ZF.^24^^,^^25^ This knowledge around IMiD/CeLMODs has led to the development of a range of tags showing various levels of efficiency and versatility in target degradation such as IKZF3-25mer,^26^ IKZF3 136–180_236-249^27^ and more recently the SuperDegron (SD).^28^

To address the limitations of the currently available protein modulation tools and better understand the degradation efficiencies of IMiD/CeLMOD based degrons, we set out to develop a robust system for targeted protein degradation by exploiting the fusion of an IMiD/CeLMOD recognition sequence to a protein of interest. Herein we present the development of the “iTAG” (Inducible and TArgeted protein deGradation), an inducible degradation TAG that elicits controlled protein degradation across targets and cell types. By systematically evaluating a matrix of 23 degron containing domains (DCDs) from various ZF and non-ZF proteins with a panel of IMiD/CeLMOD small molecules, we identified the chimeric sequence iTAG(DCD23, 60 residues), that induces acute target degradation when tagged at either N or C terminal ends. Our data also show that iTAG can be used for *in vivo* target degradation, with effective loss of target as early as 4h after a single oral administration of the thalidomide analogue, CC220. The iTAG system is not limited by the so-called hook-effect of PROTAC-based systems (ex. dTAG) and elicits more robust maximal degradation than these state-of-the-art approaches. Importantly, we show that the chimeric iTAG, as opposed to natural ZF-derived DCDs, is active in mouse cells without the need to humanize CRBN and indicate that additional murine glycine β-hairpin loop degrons may also be degraded with the appropriate thalidomide derivative. We therefore submit the iTAG system as a modular and versatile tool for protein degradation *in vitro* and *in vivo* for both human and murine models.

## Results

### Design of the DCD tags

To identify the optimal recognition motif for protein degradation with known IMiD/CELMoD agents, we sought to systematically evaluate native and chimeric degron motifs against current IMiD/CELMoD agents. Leveraging structural and sequence analysis of known CRL4^CRBN^neo-substrates (GSPT1, Ikaros, Aiolos, Eos, CK1α and ZFP91), we selected 23 DCD tags of various sizes, ranging from 10 to 197 residues in length, for experimental evaluation; these contained the glycine β hairpin loop flanked by extended amino acid sequences from the neo-substrates. These additional residues served the purpose of promoting ternary interactions between the surface of CRBN and the IMiD/CELMoD agent (Figures S1 and S2).

Three groups of DCD tags were identified. The first group of DCD tags (DCD1-6) were based on the neo-substrates GSPT1 and CK1α and provided an initial proof of concept for our tagging system. The second group of DCD tags (DCD7-20) was designed based on the C2H2 Zinc Finger neo-substrates (Ikaros, Aiolos, Eos and ZFP91). The final group of DCD tags (21–23) was based on a degron hybrid between two ZF CRBN substrates: Ikaros and ZFP91. This final group leveraged the results from the work of Sievers et al. that demonstrated that chimeric Aiolos/ZFP91 ZF had higher affinity for CRBN compared to the Aiolos or ZFP91 ZFs^25^ and that a stretch of three consecutive arginine residues within Ikaros ZF3 (R183-R185) were critical in enhancing the binding to CRBN. (Detailed structure and design of the DCD tags is described in STAR Methods section and (Figures S1 and S2).

### Selection and characterization of optimal degradation tag

**Figure 1:**
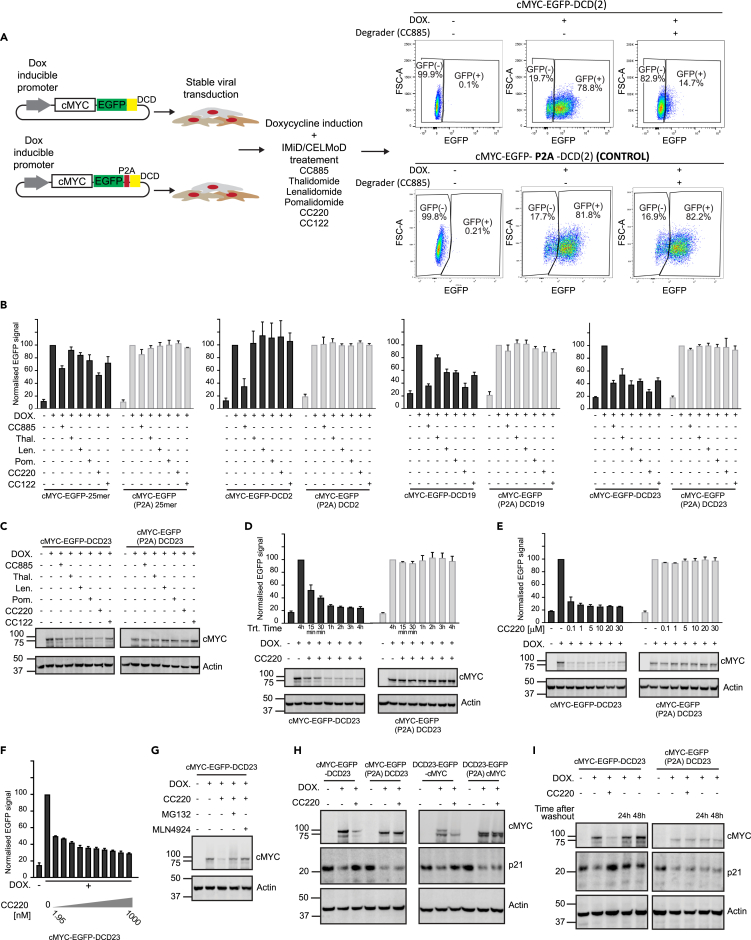
Design and experimental evaluation of the DCD tags (A) Representation of the experimental pipeline used to evaluate the different DCD constructs. The data exemplifies DCD2 treated with CC885, HMEC cells containing the cMYC-EGFP-DCD2 construct consistently showed loss of EGFP signal in the presence of CC885 agents, whereas the cells expressing cMYC-EGFP(P2A)DCD2 did not, because of the uncoupling of the DCD from the cMYC-EGFP. (B) HMEC stably expressing Dox inducible cMYC-EGFP-DCD2/19/23/25mer or their corresponding P2A controls were induced with doxycycline for 24h then treated with either DMSO or the different indicated drugs at a concentration of 10uM for 4h. Median EGFP intensity was measured in each condition using flow cytometry (3 independent experiments, normalized to the control and represented as mean ± SD). (C) HMEC stably expressing Dox inducible cMYC-EGFP-DCD23 or the corresponding P2A control were induced with doxycycline for 24h then treated with either DMSO or the different indicated drugs at a concentration of 10uM for 4h. The protein lysates were probed for cMYC levels using western blotting. (D) Cells used in (C) were induced with doxycycline for 24h then treated with either DMSO or CC-220 at a concentration of 10uM for different time points. Target loss was evaluated either by measuring EGFP median intensity using flow cytometry or western blotting for cMYC protein (3 independent experiments, normalized to the control and represented as mean ± SD). (E and F) Cells used in C were induced with doxycycline for 24h then treated with either DMSO or CC-220 at the different indicated concentrations for 4h. Target loss was evaluated as in (D) (3 independent experiments, normalized to the control and represented as mean ± SD). (G) Cells used in (A) were induced with doxycycline for 24h then treated with either DMSO or CC220 (10μM) in the presence or absence of MG132 or MLN4924 for 4h and analyzed using western blotting. (H) HMEC cells stably expressing Dox inducible N-terminally tagged DCD23-EGFP-cMYC, C-terminally tagged cMYC-EGFP-DCD23 or their corresponding P2A controls were induced with doxycycline for 24h then treated with either DMSO or CC220 (10μM) for 16h. Protein lysates were collected and probed for cMYC and p21 using western blotting. (I) Cells used in (C) were induced with doxycycline for 24h then treated with CC-220 (10μM) for 4h. Subsequently the drug was washed out, by reseeding the cells in a fresh vessel without CC-220 and left to express the construct for 24h or 48h. Protein lysates were collected and probed for cMYC and p21 using western blotting.

To systematically delineate the optimal DCD/degrader combination for targeted protein degradation, we undertook a matrix approach evaluating all 23 DCD tags against 6 small molecule degraders. First, we fused the DCD tags with Enhanced Green Fluorescent Protein (EGFP) and a target protein of interest, focusing initially on the proto-oncogene cMYC (Figure 1A). cMYC function has been extensively studied and downstream perturbations of cMYC loss are well characterised,^29^^,^^30^^,^^31^ providing a good reference to test the iTAG system. Furthermore, previous attempts to degrade cMYC using alternative approaches have resulted in sub-optimal loss of the target.^10^ To control for any potential off-target effects of the IMiD/CeLMOD agents that could indirectly affect the target of interest, we designed controls in which the specific DCD and the EGFP sequences were interspaced by a P2A ribosomal “skipping” sequence,^32^ leading to separate translation of the DCD and the target protein (Figure 1A). These constructs were then inserted into a doxycycline-inducible lentiviral vector system and stably expressed in hTERT immortalized human mammary epithelial cells (HMEC), a suitable model to assess the functional consequences of cMYC degradation. cMYC was tagged at the C-terminus end for the initial screening experiments.

We undertook a cellular screen experiment for all 23 DCD tags (Figure S3). The criteria used to select the optimal DCD candidate included: i) evidence of efficient, reproducible and robust degradation of the target protein; ii) short sequence length to avoid interference with normal protein functions; iii) evidence of effect with IMiD/CELMoD agents that do not cause direct cellular toxicity: and iv) evidence of more active degradation than that previously described for the 25-mer Aiolos (ZF2)-based degron.^26^

Flow cytometry analysis showed that the GSPT1-based DCD2 (113 aa, 13.5 kDa) led to a strong decrease in EGFP signal, but only when cells were exposed to CC885 as expected because of the substrate specificity of the different degrader compounds (Figures 1B, S3,S4, and Table S1). The potency of degradation was equivalent to the longer GSPT1-based DCD1 (197aa), whereas DCD3, which contained only the GSPT1 β-hairpin loop, and the extended DCD4 and 5 failed to induce cMYC degradation, suggesting that degradation induction required the full C-terminal β-barrel domain of GSPT1 (Figures S1 and S3). However, CC885 induced significant cellular toxicity in HMEC cells and in other cells tested (HEK293T, MDA-MB-231, Kelly), so the DCD2/CC885 pair did not represent a suitable combination for further evaluation (Figure S5). This clearly highlights the need for an appropriate IMiD/CELMoD agent in the selection of the optimal DCD tag and degrader molecule pair.

Of the native ZF based DCDs, the 131 amino acid Ikaros-based DCD19 (131 aa, 15.7 kDa) induced robust loss of cMYC-EGFP signal with all the degraders tested (CC885, thalidomide, lenalidomide, pomalidomide, CC-122 and CC220) (Figures 1B, S3, and S4). Use of chimeric DCDs improved degradation, with the chimeric Ikaros/ZFP91 DCD23 (60 aa, 7 kDa) inducing degradation at a significantly shorter length (Figures 1B, S3, and S4). CC220 was the most active protein degrader (Figures 1B and S3) and did not induce cellular toxicity (Figure S5). Loss of EGFP, observed using flow cytometry, in DCD23 indirectly indicated cMYC degradation. The loss of cMYC was subsequently confirmed by western blotting (Figure 1C). CC220 caused a significant degradation of c-MYC tagged with DCD23 within 15 min (Figure 1D), and at nanomolar concentrations (DC_50_ at 4h = 2 nM; DCmax at 4h 75%) (Figures 1E and 1F). We confirmed that the degradation was mediated by the ubiquitin-proteasome system, as co-treating the cells with CC220 and either the proteasome inhibitor MG132 or the neddylation inhibitor MLN4924, inhibited cMYC degradation (Figure 1G).

We then assessed if the iTAG-induced degradation could be used to explore the function of the protein of interest. The cyclin-dependent kinase inhibitor p21 is a well described target of cMYC transcriptional repression.^31^^,^^33^ On induction of cMYC-EGFP-DCD23 or the control cMYC-EGFP(P2A)DCD23, p21 levels decreased in HMEC cells (Figure 1H). Subsequent treatment of the cells with CC220 induced the degradation of DCD23-tagged cMYC and was accompanied by a restoration of p21 levels. In the control cMYC-EGFP(P2A)DCD23 cells, CC220 did not induce the degradation of cMYC and consequently no restoration of p21 levels was observed, thereby confirming the specificity of the effect (Figure 1H). These results indicate that iTAG can be used to explore the functional consequences of target protein degradation, with parallel confirmation of the specific molecular consequences, as they do not occur in the P2A′ skip’-containing controls.

Similarly, to the way we probed for the function of C-terminally tagged cMYC, we tested the function of N-terminally tagged cMYC (Figure 1H). On induction of DCD23-EGFP-cMYC or the control DCD23(P2A)EGFP-cMYC, p21 levels decreased in HMEC cells. Treatment with CC220 led to a restoration of p21 levels in DCD23-EGFP-cMYC cells but had no effect in the corresponding P2A control confirming the specificity of the effect observed (Figure 1H).

Another advantage of our acute protein degradation system compared to genetic approaches is the ability to rescue the protein expression after degradation in a one-step approach. This allows us to explore the downstream consequences of acute target loss and to confirm the specificity of these results by restoring target expression in the same cellular context. We tested if washing out CC220 would allow restoration of cMYC levels back to baseline. In cells expressing cMYC-EGFP-DCD23 or the control cMYC-EGFP(P2A)DCD23, treatment with CC220 leads to a decrease in the levels of cMYC-EGFP-DCD23, but not the P2A control. After CC220 removal, c-MYC levels were restored back to pre-treatment levels and, importantly, p21 repression was also restored (Figure 1I). These results show that iTAG allows for a substantial control over target modulation, thus enabling the functional study of a target protein by inducing its degradation followed by washout experiments that enable rescue of the phenotype in a one-step assay. This together with the parallel P2A control enables the specific modulation of target function.

Altogether, DCD23 with CC220 were selected as the optimal pairing for further characterization.

### DCD23 efficiently engage CRBN/DDB1 *in vitro*

To test whether the degradation of DCD23, discovered using the HMEC system, was due to a direct interaction with CRBN induced by IMiDs/CELMoDs, we developed two types of *in vitro* assays which allowed control over all the components, and used them to quantify the interactions.

First, we wanted to confirm that the IMiD/CELMoDs used in our study were effectively binding to CRBN and to estimate their binding affinities. We used a fluorescence polarisation (FP) assay based on a sulfocyanine5 fluorescently labeled IMiD probe (Figure S6A).^34^ We first demonstrated the binding of the probe to CRBN/DDB1 complex by FP and measured a Kd of 36 nM. We then assessed the relative affinities of the various IMiDs/CELMoDs for the DDB1-CRBN complex by measuring their ability to displace the FP probe from the protein complex and showed that they were all able to bind to CRBN, with IC_50s_ spanning from 0.048 to 1.208 μM; notably CC220 was the strongest binder at an IC_50_ of 0.048 μM (Figure S6B and Table S2).

Second, we developed a time-resolved fluorescence resonance energy transfer (TR-FRET) assay based on an N-terminally sulfo-cyanine5 fluorescent-labelled Aiolos peptide probe, to estimate the binding affinities of our DCDs to the complex formed between IMiDs/CELMoDs and CRBN-DDB1 (Figure S6C).^35^ We first confirmed the binding of the probe to a pre-formed DDB1-CRBN+IMiD complex in presence of the different IMiDs/CELMoDs used in this study and found that Kd values ranged from 0.11 to 1.27 μM (Figure S6D and Table S2). We then assessed the relative binding affinities of DCD23 for the different DDB1-CRBN+IMiD complexes by measuring their ability to displace the Aiolos probe and determined the Ki for each compound. DCD23 bound to the various DDB1-CRBN+IMiD complexes with different affinities depending on which IMiD/CELMoD was used; with the most potent being CC220 with a Ki of 0.015 μM (Figure S6E and Table S2).

Our *in vitro* data confirmed the direct binding of the DCD23 to the complexes formed between CDBN-DDB1 and various IMiDs/CELMoDs with high affinity. These data also provided further evidence that DCD23 binds to the DDB1-CRBN complex with the highest affinity when paired with CC220 and constitutes the optimal iTAG system.

### iTAG(DCD23) can target both cytoplasmic and nuclear proteins for degradation

In order to assess the versatility of iTAG(DCD23) as a tool for protein degradation, we tested the ability to induce the degradation of targets in various cellular compartments (cytoplasm and nucleus). We also tested iTAG-induced degradation of N- or C-terminally tagged target proteins. As an example of a cytoplasmic/membrane protein, we chose the oncogenic form of KRAS, containing the G12V mutation (KRAS^[G12V]^). In addition to cMYC, we selected the histone methyltransferase EZH2 as an example of a nuclear protein that is also present within the large multicomponent polycomb repressive complex.

**Figure 2:**
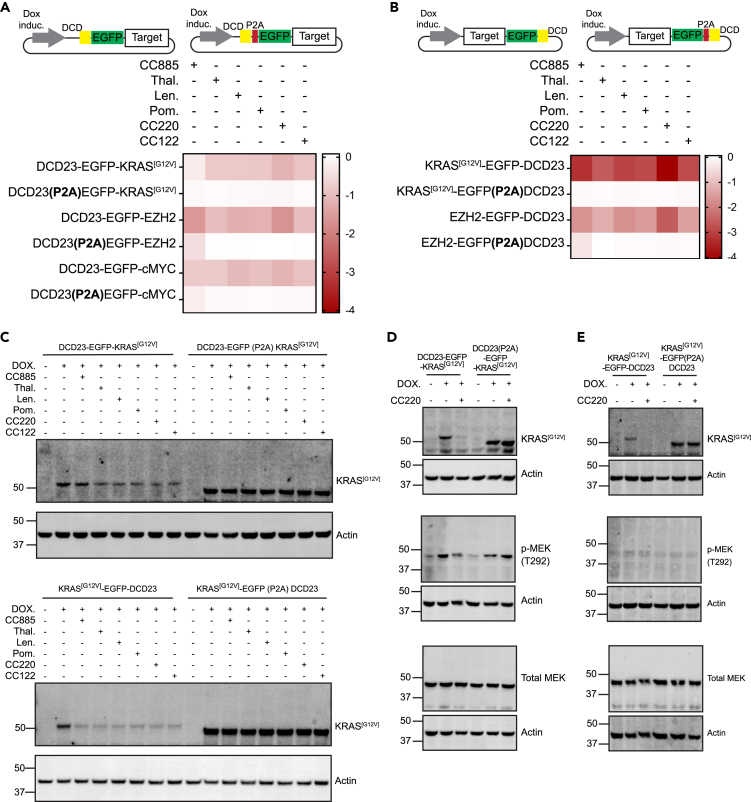
Degradation of cytoplasmic and nuclear targets using iTAG (A) HMEC cells expressing N-terminally tagged DCD23-EGFP-KRAS^[G12V]^, DCD23-EGFP-EZH2, DCD23-EGFP-cMYC or their corresponding P2A control were induced with doxycycline for 24h then treated with either DMSO or the different indicated drugs at a concentration of 10uM for 4h. Median EGFP intensity was measured in each condition using flow cytometry and normalized values were represent in the Log2 Fold change relative to the DMSO control. (B) HMEC cells expressing Dox inducible C-terminally tagged KRAS^[G12V]^-EGFP-DCD23, EZH2-EGFP-DCD23 or their corresponding P2A control were induced with doxycycline for 24h then treated with either DMSO or the different indicated drugs at a concentration of 10uM for 4h. Median EGFP intensity was measured in each condition using flow cytometry and normalized values were represent in the Log2 Fold Change relative to the DMSO control. (C) HMEC cells expressing N-terminally tagged DCD23-EGFP-KRAS^[G12V]^, C-terminally tagged KRAS^[G12V]^-EGFP-DCD23 or their corresponding P2A control were induced with doxycycline for 24h then treated with either DMSO or the different indicated drugs at a concentration of 10uM for 4h. The protein lysates were probed for KRAS^[G12V]^ using western blotting. (D and E) HMEC cells expressing N-terminally tagged DCD23-EGFP-KRAS^[G12V]^ or their corresponding P2A control in (D) and C-terminally tagged KRAS^[G12V]^-EGFP-DCD23 or their corresponding P2A control in (E) were induced with doxycycline for 24h then treated with either DMSO or CC220 at a concentration of 10uM for 48h. The protein lysates were probed for KRAS and *p*-MEK1(T292) using western blotting.

We tagged the proteins with DCD23 constructs, fused either to their N-terminus or C-terminus (Figures 2A and 2B). We stably transduced HMEC cells with these constructs and assessed their degradation. Cellular treatment with all degraders led to a decrease in the EGFP signal of the N and C-terminally tagged KRAS^[G12V]^ and EZH2 (Figures 2A and 2B). These data also confirmed CC220 as the most active degrader of DCD23 tagged targets and confirmed that the iTAG can induce degradation of both nuclear and cytoplasmic protein, fused with the tag at both N- and C-terminal ends.

We then analyzed the functional consequences of KRAS^[G12V]^-induced degradation. We confirmed the degradation of the fusion constructs by western blotting for KRAS^[G12V]^ (Figure 2C). Subsequently, we analyzed the functional consequences of KRAS^[G12V]^-induced degradation. We chose to probe for MEK1 (T292) phosphorylation as a downstream marker of KRAS activation in cells expressing N-terminally tagged DCD23-EGFP-KRAS^[G12V]^ or in cells expressing the C terminally tagged KRAS^[G12V]^-EGFP-DCD23. Doxycycline induction of N-terminally tagged KRAS^[G12V]^ led to an increase of MEK1 phosphorylation (T292) (Figure 2D), but this increase was not observed with C-terminally tagged KRAS^[G12V]^ (Figure 2E), which is most likely due to the disruption of the farnesylation of its C-terminal CAAX motif by the fusion, resulting in interference with this function.^36^^,^^37^ Following CC220 treatment, N-terminally tagged KRAS^[G12V]^ was degraded, with a concomitant decrease in MEK1 (T292) phosphorylation. MEK1 (T292) phosphorylation levels remained unaltered in cells transduced with the corresponding P2A control constructs (Figure 2D). These data whilst confirming the versatility of the iTAG system in inducing protein degradation when fused at both N- and C-terminal ends, also indicate that careful evaluation is needed in defining the end of the protein to be tagged for functional studies, which should be related to the target of interest rather than the DCD23 tag *per se*.

### Comparative evaluation of iTAG against current degron systems

Among the degradation tags currently available, the recently developed dTAG has become one the most widely adopted systems in the scientific community.^15^^,^^38^ In the dTAG system, a mutant FKBP12^[F36V]^ protein is fused to the target protein of interest and degradation is induced using a PROTAC compound (dTAG-13) targeting the mutant FKBP12^[F36V]^ to the E3 ligase CRL4^CRBN^ for ubiquitination. The broad adoption of the dTAG system has enabled to uncover numerous biological insights.^39^^,^^40^^,^^41^^,^^42^ Given these merits, we wanted to compare the degradation efficiency of our iTAG system to the well-established dTAG.

**Figure 3:**
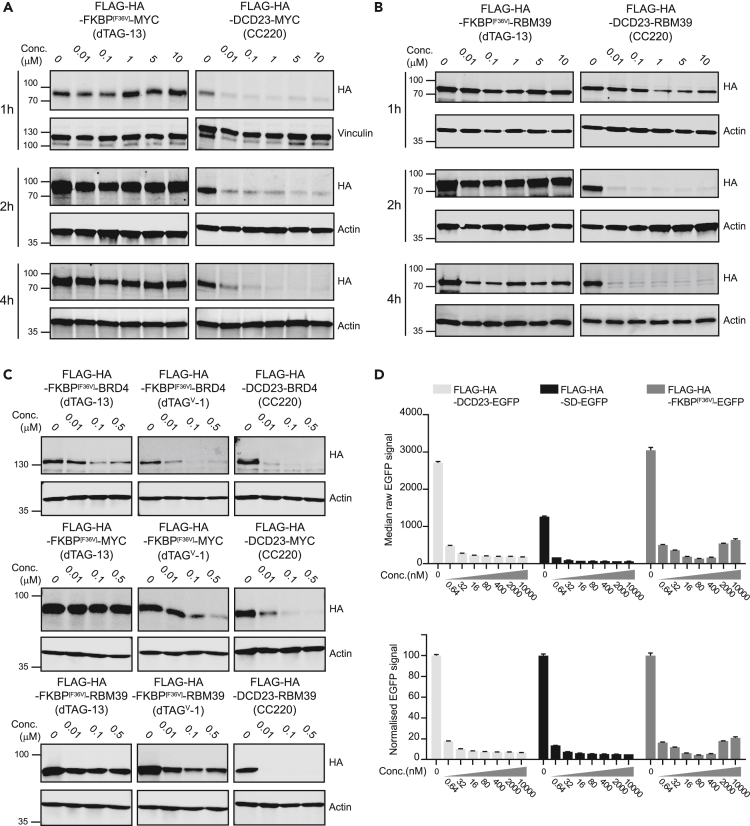
Compared degradation of iTAG and dTAG fused proteins (A and B) SUM159 cells stably expressing N-terminally tagged cMYC (A) or RBM39 (B) with dTAG (Flag-HA-FKBP12^[F36V]^) or iTAG (Flag-HA-DCD23) were treated with dTAG-13 or CC220 respectively for 1, 2 or 4h at the indicated doses. The protein lysates were probed for HA using western blotting. (C) SUM159 cells stably expressing N-terminally tagged BRD4, cMYC or RBM39 with dTAG (Flag-HA- FKBP12^[F36V]^) or iTAG (Flag-HA-DCD23) were treated for 4h with dTAG-13 or dTAG^V^-1 for dTAG or CC220 for iTAG at the indicated doses. The protein lysates were probed for HA using western blotting. (D) SUM159 cells stably expressing N-terminally tagged EGFP with dTAG (Flag-HA- FKBP12^[F36V]^), SuperDegron (Flag-HA-SD) or iTAG (Flag-HA-DCD23) were treated for 4h with dTAG-13 for dTAG or CC220 for SD and iTAG at the indicated doses. Median EGFP intensity was measured in each condition using flow cytometry. (3 independent experiments, represented as mean ± SD).

We first compared the kinetics of the acute degradation of N-terminally tagged cMYC and RBM39 (RNA-binding protein 39) using the dTAG or iTAG system, for which degradation was induced using the PROTAC dTAG-13 or CC220, respectively. The fusion constructs were stably expressed in SUM159 cells and loss of protein was determined by western blotting. At the early time points considered, dTAG induced moderate degradation only after 4h of treatment whereas iTAG led to the degradation of both cMYC and RBM39 as early as 1h after treatment. Importantly, iTAG degraded the targets across the whole range of concentrations tested (0.01–10μM), whereas dTAG’ induced degradation was less pronounced or even not observed at high concentrations, most likely as a result of the well described hook effect associated with PROTACs (Figures 3A and 3B).^43^

Subsequently, we evaluated the extent of degradation induced by the two tagging systems. For this purpose, we tested targets present in different subcellular localizations at a longer time point (24h): RBM39 (-nuclear 59.3kDa-), CDK9 (Cyclin-dependent kinase 9 -nuclear 42.7kDa-), MAPK9 (Mitogen-activated protein kinase 9 -nuclear 48.1 kDa-), HPRT1 (Hypoxanthine Phosphoribosyltransferase 1-cytoplasmic 24.5kDa-), SMS (Spermine synthase –cytoplasmic 41.2kDa-), and MAVS (Mitochondrial antiviral-signaling protein -mitochondrial 56.5kDa-) (Figure S7). Although discrepancies were observed in the expression levels of the two systems, both tags induced the degradation of all the proteins tested, with iTAG consistently inducing a more effective degradation of the targets even at low nanomolar CC220 concentrations.

The dTAG system has also been adapted to direct targets to the E3 CRL2^VHL^ using the PROTAC dTAG^V^-1^16^. This variant was shown to be more effective compared the original CRL4^CRBN^ system.^16^ We evaluated the degradation N-terminally tagged cMYC and RBM39 dTAG^V^-1, we included BRD4 as a control as it was reported to be a well degraded target in the initial dTAG optimization (Figure 3C).^15^ For all three targets, dTAG-v1 induced a more robust degradation compared to dTAG-13, however iTAG-induced degradation appeared to be superior to the two dTAG variants.

As we were finalizing our study on iTAG, a similar IMiD based degron was publish by the Ebert group.^28^ The SuperDegron (SD) a 60 amino acid tag based on a chimera of Aiolos/ZFP91 was used to generate degradable chimeric antigen receptors (CAR).^28^ To compare the degradation profile, we stably expressed N-terminally tagged EGFP with either iTAG, SD or FKBP^[F36V]^ in SUM159 cells and analyzed the degradation using flow cytometry. iTAG and FKBP^[F36V]^ tagged constructs were expressed at similar level, but SD construct had a significantly lower expression (approx. 60% less) despite using the same expression vector (Figure 3D). Based on normalized value the different tags showed equivalent DC_50_s (iTAG = 0.39 nM, SD = 0.37 nM, FKBP12^[F36V]^ = 0.38 nM) and DCmax (iTAG = 93%, SD = 95%, FKBP12^[F36V]^ = 95%) although the dTAG system exhibited some hook effect at high concentration (Figure 3D).

These results further confirm iTAG as a versatile tool for the acute and efficient protein degradation of a wide range of targets, but also suggest caution when comparing different tagging systems as any conclusions should take in consideration baseline protein expression level and cellular context, as was highlighted in a recent systematic degron comparison study.^44^

### *In-vivo* degradation of iTAG fused proteins

To evaluate the iTAG system *in vivo*, we assessed its ability to degrade target proteins in human tumor xenografts. MDA-MB-231 breast cancer cells were generated to stably express the firefly luciferase (FLuc) construct tagged with EGFP-DCD23 under doxycycline inducible promoter control (Figure S8A). *In vitro* treatment of these cells with CC220 led to a decrease in both the luciferase and EGFP signal within 3h and at concentrations as low as 0.05μM (Figures S8A and S8B). The loss of the fusion construct on CC220, but not of its P2A control, treatment was also confirmed by western blotting (Figure S8C).

**Figure 4:**
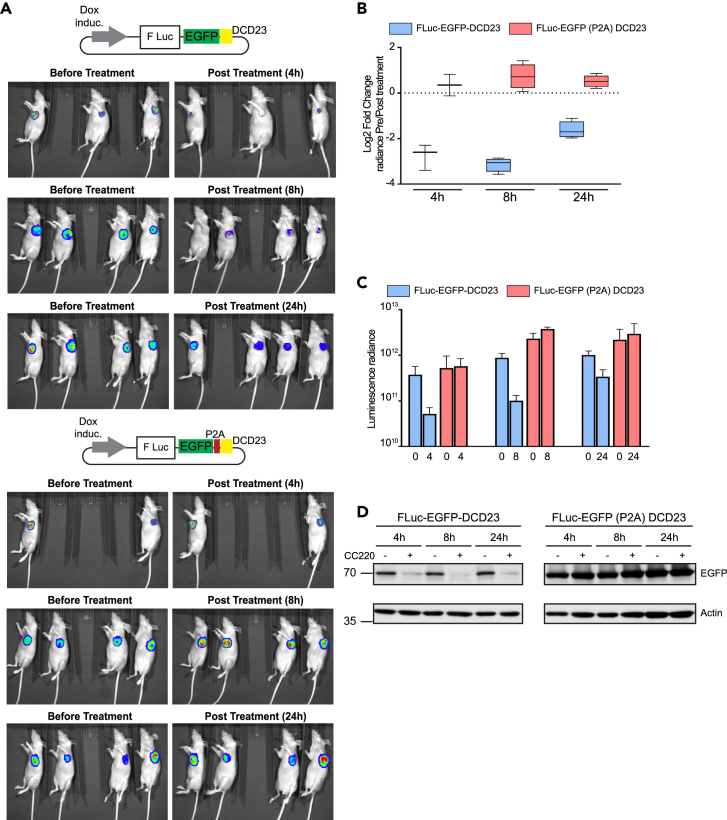
*In-vivo* degradation using iTAG (A) Bioluminescence images of orthotopic tumors formed from MDA-MB-231 cells stably expressing Dox. inducible FLuc_EGFP_DCD23 or FLuc_EGFP(P2A)DCD23 before (pre) and after (post) systemic administration of 30 mg/kg CC-220 by oral gavage. (B and C) Quantification of bioluminescence signals from mice represented in A, represented in either Log2 fold change pre/post treatment in (B) or in absolute values (C) (data from at least 2 animals, represented as mean ± SD). (D) Tumors from mice treated in (A) were lysed and probed for FLuc using western blotting.

MDA-MB-231(FLuc-EGFP-DCD23) and MDA-MB-231(FLuc-EGFP P2A DCD23) control cells were injected orthotopically in the mammary fat pads of immunocompromised mice. Once tumors reached 300–350 mm^3^ in volume, FLuc expression was induced using doxycycline. The mice bearing MDA-MB-231(FLuc-EGFP-DCD23) or MDA-MB-231(FLuc-EGFP P2A DCD23) tumors were randomized to receive 30 mg/kg CC220 or vehicle. Luminescence was measured before and 4h, 8h or 24h after CC220 or vehicle administration. Luminescence intensity rapidly decreased by a Log2 fold change of −2.76 and −3.13 at 4h and 8h, respectively (Figures 4A–4C and S8D). Loss of the fusion construct on CC220 treatment was confirmed using western blotting (Figure 4D).

At 24h post CC220 administration, FLuc-EGFP-DCD23 luminescence remained lower despite residual plasma CC220 levels were in the nanomolar range (Log2 fold change of −1.63) (Figures 4A–4C and S8E). This was also seen on western blot (Figure 4D), suggesting that even nanomolar plasma concentration of CC220 is sufficient to maintain target degradation *in vivo*. Together, these data collectively show that iTAG allows rapid and sustained degradation of tagged proteins *in vivo*.

### iTAG induces the degradation of targets in murine cells

**Figure 5:**
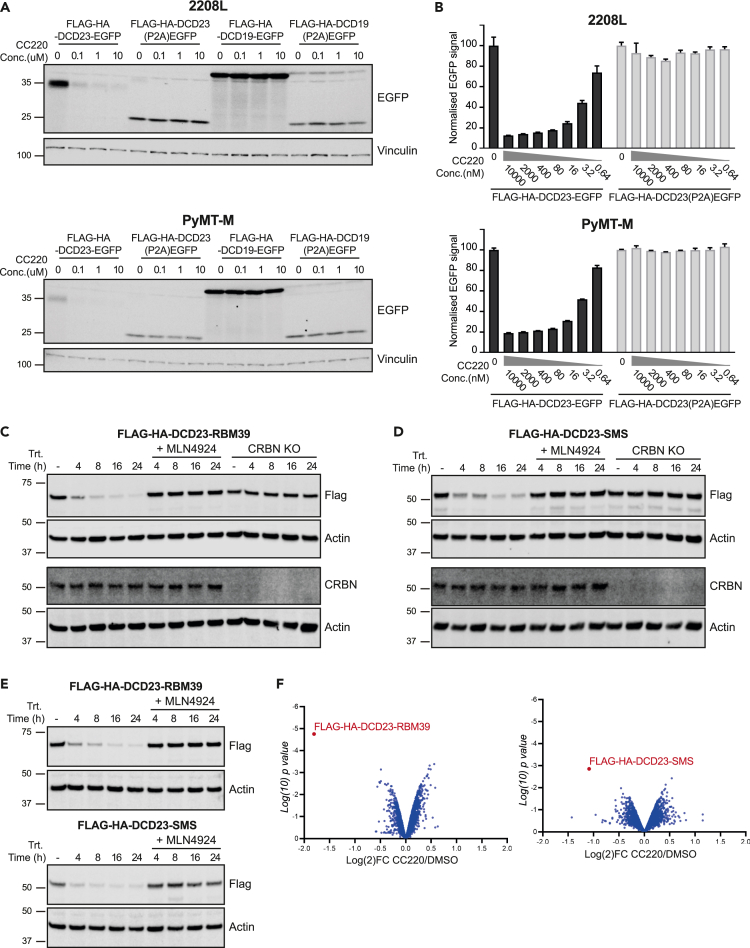
iTAG induced degradation in murine cells (A) 2208L or PyMT-M cells stably expressing Flag-HA-DCD19-EGFP, Flag-HA-DCD23-EGFP or their corresponding P2A control were treated with CC220 for 24 h at the indicated doses. The protein lysates were probed for EGFP using western blotting. (B) 2208L or PyMT-M cells stably expressing Flag-HA-DCD23-EGFP or its corresponding P2A control were treated with CC220 for 24 h at the indicated doses. EGFP signal was then measured using flow cytometry. (3 independent experiments, normalized to the control and represented as mean ± SD). (C and D) CT26 or CT26 CRBN KO cells stably expressing Flag-HA-DCD23-RBM39 C or Flag-HA-DCD23-SMS D were treated with 10uM CC220 for the different time point in presence of absence of MLN4924 and analyzed using western blotting. (E) AT3 cells stably expressing Flag-HA-DCD23-RBM39 or Flag-HA-DCD23-SMS were treated with 10uM CC220 for the indicated time point in presence of absence of MLN4924 and analyzed using western blotting. (F) Proteomes of CT26 stably expressing Flag-HA-DCD23-RBM39 or Flag-HA-DCD23-SMS treated with 10uM CC220 for 8h analyzed using quantitative proteomics. Data are plotted as Log2 fold change relative to DMSO versus Log10 of p value.

Given the high degradation efficiency of DCD23 in human cells, we sought to test the applicability of this system in murine cells. We expressed EGFP constructs N-terminally tagged with either DCD19 or DCD23 with their corresponding P2A controls in murine mammary cancer lines (2208L and PyMT) and tested their degradation with CC220. The DCD19 tagged constructs where not degraded after CC220 treatment, which was expected given that DCD19’s sequence was extracted from native IKZF1 sequence. Surprisingly, DCD23 tagged EGFP was efficiently degraded on CC220 treatment in both cell lines (Figure 5A).

Given these unexpected results, we characterized the kinetics of iTAG (DCD23) induced degradation in murine cells using flow cytometry and found that iTAG (DCD23) induced loss of EGFP signal at nanomolar concentrations of CC220 (DC_50_ = 2.7nM and 4.3 nM at 24h in 2208L and in PyMT respectively) (DCmax = 88% and 81% at 24h in 2208L and in PyMT respectively) and as early as 1h post treatment (Figures 5B and S9A). As an additional validation of iTAG (DCD23) induced degradation, we tested the same constructs in murine colorectal carcinoma cells (CT26) where we saw similar loss of EGFP with a panel of IMiD/CeLMODs (Figure S9B).

We then tested the degradation of RBM39 (nuclear) and SMS (cytoplasmic) N-terminally tagged with iTAG (DCD23) in CT26 and AT3 (murine mammary cancer lines) (Figures 5C–5E). We found that the targets were degraded in both cells lines and that the degradation was dependent on the presence of CRBN (Figures 5C–5E). To further understand the degradation profile in an unbiased manner, we used quantitative TMT (Tandem Mass Tagging) multiplexed proteomics in CT26 cells and found that CC220 treatment induced the robust degradation of iTAG (DCD23) tagged RBM39 and SMS without any detected off-targets (Figure 5F).

Previous studies have reported that IMiDs/CELMoDs do not induce the degradation of known neo-substrates (IKZF1, IKZF3, CK1α, ZFP91) in mice because of the Val388 in human CRBN being substituted for Ile391 in murine CRBN which causes steric hindrances preventing the recruitment of neo-substrates.^45^^,^^46^^,^^47^ Consistent with this data, tagging with sequence extracted from the native IKZF1 in DCD19 did not induce degradation in murine cells. On the other hand, DCD23, which has chimeric structure that differs from the native neo-substrates, does effectively induce degradation of fused targets in mouse cells. This further expands the usability and versatility of the iTAG system to both human and murine models.

### Exploring further degron opportunities in murine cells

After showing that iTAG was active in mouse cells, we explored whether more potential degrons were active in the murine context. We treated CT26 mouse cells with commercially available thalidomide derivatives CC90009,^48^ StJude6989,^49^ FTFP2216^50^ and profiled the cellular proteomes using quantitative multiplexed proteomics.

**Figure 6:**
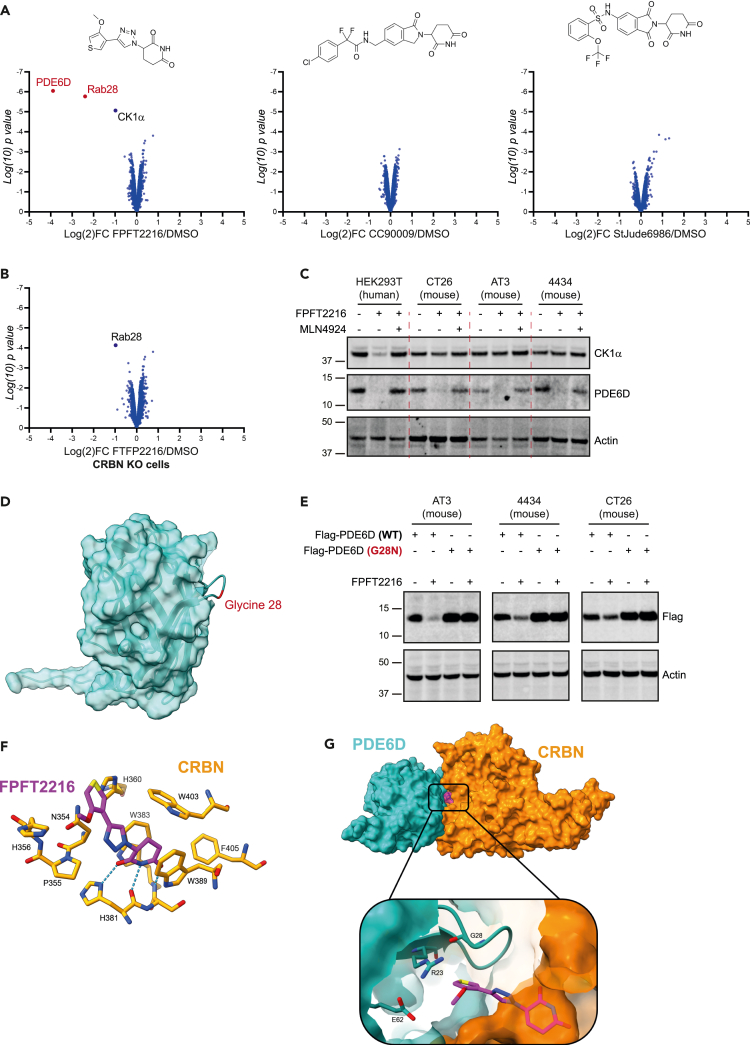
FPFT2216 induces the degradation of PDE6D in murine cells (A) Proteomes of CT26 cells treated with 10uM of the indicated compounds for 24h and analyzed using quantitative proteomics. Data are plotted as Log2 fold change relative to DMSO versus Log10 of p value. (B) Proteome of CT26 CRBN KO cells treated with 10uM of FPFT2216 for 24h analyzed and plotted as in (A). (C) HEK293T, CT26, AT3 and 4434 cells treated with 10uM of FPFT2216 for 24h in presence of absence of MLN4924. Lysats were analyzed using western blotting. (D) Structural representation of PDE6D with the critical Glycine 28 highlighted in red. (E) CT26, AT3 and 4434 cells transiently expressing WT or G28N mutant Flag-PDE6D treated with 10uM of FPFT2216 for 16h. (F) Docking model for FPFT-2216 (in magenta) in the binding site of mouse CRBN (in orange). H-bond interactions are shown as cyan dashes. (G) Binding model for the ternary complex formed by mouse CRBN (in orange), mouse PDE6D (in cyan) and FPFT-2216 (in magenta), showing the Gly28 loop close to the molecular glue.

We found that CC90009 and StJude6989 which are known GSPT1 degraders in human cells had no impact in CT26. However, FPFT2216 induced a strong downregulation of PDE6D, Rab28 and to a much lesser extent CK1α (Figure 6A). These effects of FPFT2216 were CRBN dependent as we did not observe them in CT26 CRBN KO cells (Figure 6B). Rab28 has been described as a client of PDE6D and hence its downregulation could be potentially secondary to PDE6D degradation,^51^^,^^52^ although some downregulation was observed in the CRBN KO cells and suggests a more complex activity (Figure 6B). We focused on PDE6D and CK1α for western blot validation, using human HEK293T cells as a control, we found that FPFT2216 induced the degradation of PDE6D in multiple mouse cell lines (AT3, 4434 and CT26) but not the degradation of CK1α, which was only degraded in human cells (Figure 6C).

After validating PDE6D degradation, we progressed to identify the critical degron site on the protein. We hypothesized that PDE6D binding to CRBN would require a Gly loop motif and used known structural features of IMiD degrons to map this site in PDE6D (see STAR Methods for details). Our modeling suggested the Glycine28 loop as a potential degron (Figure 6D). We mutated this Gly28 to Asparagine to disrupt the interaction with CRBN and expressed the Flag tagged construct in murine cells. In contrast the WT PDE6D constructs, the G28N mutant were not degraded by FPFT2216 treatment confirming the importance of the Gly28 loop as the critical degron for PDE6D degradation (Figure 6E).

We then worked to better understand the recruitment of PDE6D to murine CRBN using *in-sillico* modeling. A docking model for FPFT2216 bound to the thalidomide binding domain of mouse CRBN was generated, showing the glutarimide ring of FPFT2216 located in the tri-Trp pocket of CRBN and involved in H-bond interactions with His381 and Trp383 as typically observed with IMiDs (Figure 6F). Unconstrained protein-protein docking simulations of mouse PDE6D against the generated model led to the identification of a pose showing the Gly28 loop in proximity to FPFT2216 (Figure 6G). Molecular dynamics simulations, used to confirm the stability and refine the predicted ternary complex conformation, highlighted how Gly28 is consistently found at close distance from FPFT2216 across the MD trajectories. The average distance between the Gly28-Cα and the closest atom of FPFT2216 was found to be 3.3 Å (Figures S10A and S10B), similar to the one observed for other neo-substrate Gly degrons in crystallographic complexes formed with IMiDs: e.g., 3.2 Å in the CRBN:POM:IKZF1 complex (Gly151 in PDB 6H0F), 3.5 Å in the CRBN:CC-90009:GSPT1 complex (Gly574 in PDB 6XK9), 3.5 Å in the CRBN:LEN:CK1α complex (Gly40 in PDB 5FQD). The prediction is thus in line with the experimental finding of the G28N mutant interfering with the PDE6D recognition and degradation induced by FPFT2216. Of interest, this model potential explains how PDE6D downregulation is observed in murine cell lines as we observe only weak contacts with the mouse CRBN Isoleucine391 (Figure S10C).

This data shows that further murine degrons are available for degradation with the appropriate thalidomide derivative, thus providing tools for studying protein function in murine systems.

## Discussion

We aimed to discover and develop a tagging system for the inducible degradation of proteins that was simple, robust and versatile with the ability to degrade a broad range of targets both *in vitro* and *in vivo*.

We deployed an empirical approach to investigate DCD tags, all derived from known CRL4^CRBN^ neo-substrates paired with commercially available IMiD/CELMoD agents. Our approach has led to the identification of the iTAG system a combination of DCD23 a 7 kD tag, with a chimeric degron containing sequences derived from Ikaros and ZFP91 and the molecular glue, CC220 (Iberdomide), a potent thalidomide analogue discovered for its activity in lenalidomide-resistant lymphoma cell lines.^53^^,^^54^ Iberdomide is currently in early clinical trials (phase II trials for lymphomas/multiple myelomas NCT02773030, NCT03310619). Given the well described activity IMiD/CELMoD agents in multiple myelomas cells, we strictly restricted the application of this tag to targets in non-haematological cells where we validated that IMiDs/CELMoDs do not induce cytotoxicity. In addition, given the pleiotropic effects IMiDs/CeLMODs in degrading glycine loop containing ZF proteins,^24^^,^^25^^,^^46^ we have included and recommend the use of P2A constructs to control for off-target effects in human cells (Figure S11).

Using our empirical approach to define optimal tags for targeted protein degradation we were able to explore some core concepts for neo-substrate recruitment to CRBN. The IMiDs/CELMoDs have the ability to induce the degradation of target proteins that contain a degron, consisting of a β-hairpin loop with a sentinel glycine residue.^20^^,^^24^^,^^47^^,^^55^ The most comprehensively defined neo-substrates for these agents: Ikaros/Aiolos; CK1α; GSPT1 and ZFP91 all have such a β -hairpin loop but do not show any consistent sequence homology that defines interactions with CRBN apart from the glycine residue. Instead, the different classes of neo-substrates create additional ternary protein-protein or protein-degrader interactions involving residues outside of the degron, where the protein folds are different for each class of neo-substrate proteins. The DCD3 and DCD6 tags (10 and 16aa residues respectively, each containing minimal β-hairpin loops derived from the neo-substrates GSPT1 and CK1α) did not induce degradation of the target protein of interest whereas the 25mer tag derived from Aiolos ZF2 did induce partial degradation. This difference can potentially be explained by the location of the ternary interactions enabling DCD tag-, compound- and CRBN-mediated interactions. For CK1α and GSPT1, the residues necessary for these ternary interactions are located a distance away from the β-hairpin loop in the neo-substrate protein and indicate the need for additional binding domains or protein folds that enable interaction with CRBN and/or the compound (Figure S2). Therefore, despite containing the minimal β-hairpin loop, it is impossible to derive a short and efficient DCD based on these proteins. On the other hand, for ZF-containing DCD tag, the degron β-hairpin is immediately followed by an α-helix within the zinc finger, which facilitates interactions with residues at the surface of CRBN (Figure S2). It should be noted that though a 25mer minimal ZF motif is sufficient to promote the degradation of a fusion target, optimal degradation can only be achieved if additional residues are added, notably from the ZF following the one containing the degron, enabling supplementary ternary interactions between CRBN and the DCD tag. These observations are in accordance with the concept of cooperativity described by Ciulli’s group and recently mathematically modeled for molecular glues.^56^^,^^57^ Molecular glues such as the IMiD/CELMoD agents overcome the energy barrier for initial ternary complex formation (enabled by the minimal β-loop containing degron), and there then exists further potential to enhance the stability and affinity of the ternary complex via the creation of supplementary protein-protein interactions. Of note, the stability of the ternary complex and subsequent ubiquitin mediated degradation of the target protein will depend not only on the sequence of the tag but also on the chemical structure of the molecular glue and on the cellular context.^58^

Our approach has led to the identification of DCD23, a chimeric degron sequence containing a central Zinc-Finger derived from IKZF1 and ZFP91, flanked with parts of IKZF1 ZF1 and 3. This chimeric DCD tag caused greater target protein degradation than the non-chimeric degron DCDs of similar size and corresponding origin (Ikaros only: DCD18 -60aa-) and similar degradation to non-chimeric degron of larger size (Ikaros only: DCD19 -131aa-) (Figures S3 and S12), suggesting that stronger core interaction can compensate for the absence of extended surface contacts. The DCD23 tag, contains 3 consecutive arginine residues located within Ikaros’ ZF3 β-hairpin loop, which have been shown through mutational analysis to impart high affinity binding to the CRBN-DCD complex.^25^ Of interest, DCD23 led to a similar degradation as the Super Degron (IKZF3+ZFP91) that only contains 2 consecutive arginine residues (Figure S12).

A range of inducible degradation systems have been recently developed, including tags derived from IMiD degrons.^15^^,^^16^^,^^26^^,^^27^^,^^28^ Our study provides a systematic evaluation of different IMiD degrons and a comparison with leading PROTAC based systems. The dTAG system, which allows us to exploit both CRL4^CRBN^ and CRL2^VHL^ mediated via a PROTAC approach has been adopted as an important reagent for studying protein degradation and has been extensively used in the functional characterization of protein function.^39^^,^^40^^,^^41^^,^^42^ Recent work from the Broad Institute comparing various degron systems, including a 60 aa Aiolos degron equivalent to our DCD12 (which was less efficient than our DCD23), showed the versatility of the IMiD based degron compared to other degradation systems. Our direct comparison of the degradation suggests that iTAG induces more rapid and effective degradation than dTAG (both with dTAG-13 and dTAG^V^-1) across a range of drug concentration without the limiting hook effect associated with using a PROTAC.^43^ This advantage is particularly important for functional studies as it enables to explore a broader range of dose response conditions and simplifies the dose optimization for *in vivo* studies. The smaller size of the tag [iTAG; 7kDa v dTAG; 12kDa] combined with the excellent PK properties of CC220^59^ are also important features of the system.

Finally, a major limit to IMiD based degrons has been the lack of activity in murine cells.^44^^,^^46^ We show that iTAG, in contrast to native sequences, is active in mouse cells without the need for CRBN humanization and induces a specific degradation of nuclear and cytoplasmic proteins without off-target effects. This unexpected observation considerably expands the application of iTAG and could be attributed to either the improved efficiency of iTAG compared to native sequences and/or a different binding mode that overcomes the polymorphism on mouse CRBN. Furthermore, we show that mouse CRBN can be hijacked using an alternative thalidomide derivative in a mechanism that depends on Glycine loop degrons. These empirical observations will enable the further development of mouse degradation system based on specific thalidomide derivatives.

### Limitations of the study

- iTAG is part of a growing number of IMiD based degrons tags that are currently used for acute modulation of targets. In this study we performed a systematic evaluation of various tags, but have not evaluated all of the possible degrons available (such as IKZF3 136-180_236-249^27^) as we found that iTAG provided sufficient efficiency and versatility.
- IMiDs/CELMoDs have activity in a number hematological malignancies and have well described pleiotropic degradation profiles in human cells.^24^^,^^25^^,^^46^ This restricts IMiD based degrons tags to non-hematological cells and emphasizes the need for controls such as using the P2A system. Furthermore, proteomic profiling of IMiD degradation in the cells of interest would help better understand off targets in context.
- The study used stable overexpression system to discover, validate and benchmark iTAG. Application to a target of interest should preferably use endogenous knock-in to better capture the biology of the target.
- Finally, this work provides evidence showing the activity of murine CRBN using thalidomide derivatives. More work will be needed to understand the activity of iTAG in mouse cells (such as *in-vitro* binding and structural determination) and to identify novel putative murine neo-substrates.

## STAR★Methods

### Key resources table

**Table:** 

REAGENT or RESOURCE	SOURCE	IDENTIFIER
**Antibodies**		

Anti-cMYC Y69	Abcam	ab32072
Anti-KRAS (G12V) clone D2H12	Cell Signaling Technologies	#14412
Anti-Phospho MEK-1 (T292)	Abcam	ab76314
Anti-beta Actin	abcam	8226
Anti-beta Actin	Sigma Aldrich	A1978
Anti-p21 clone 12D1	Cell Signaling Technologies	2947S
Anti-EGFP	Invitrogen	A11122
Anti-Vinculin	Sigma Aldrich	V9131
Anti-HA	Cell Signaling Technologies	3724
IRDye® 680LT Goat anti-Mouse IgG	Licor	926–68020
IRDye® 800CW Goat anti-Rabbit IgG	Licor	926–32211

**Bacterial and virus strains**		

NEB® 5-alpha Competent *E. coli*	New England Biolabs	C2987H
BL21-AI™ One Shot™ Chemically Competent*E. coli*	ThermoFischer Scientific	C607003

**Chemicals, peptides, and recombinant proteins**		

Polybrene Infection/Transfection Reagent	Sigma Aldrich	TR-1003-G
Doxycycline hyclate	Sigma Aldrich	D5207
DAPI Solution	Life Technologies	62248
Lipofectamine™ 3000 Transfection Reagent	ThermoFischer Scientific	L3000001
NuPAGE MES SDS running buffer	ThermoFischer Scientific	NP0002
Thalidomide	Abcam	ab120032
Lenalidomide	Abcam	ab142129
Pomalidomide	Abcam	HY-10984
Avadomide/CC122	MedChemExpress	HY-100507
Sf-900™ III SFM	ThermoFischer Scientific	12658019
cOmplete™ULTRA Tablets	Sigma Aldrich	5892953001

**Critical commercial assays**		

Pierce BCA Protein Assay Kit	ThermoFischer Scientific	23225
Lenti-XTM GoStixTM kit	Takara Bio	631281
CellTiter-Blue® Cell Viability Assay	Promega	G8080
NuPAGE 10% Bis-Tris Midi electrophoresis gels	ThermoFischer Scientific	WG1202BOX
Bio-Rad Protein Assay Kit II	Bio-rad	5000002
TMTpro™	ThermoFischer Scientific	A44520

**Deposited data**		

Proteomics	PRIDE	PXD038299

**Experimental models: Cell lines**		

Human: h-Tert Immortalised HMEC (female)	Laboratory of Prof. Martin Eilers	N/A
Human: SUM159 (female)	BioIVT	SUM-159PT
Human: HEK293T (embryonic female)	ATCC	ATCC CRL-3216
Human: MDA-MB-231 (female)	ATCC	ATCC HTB-122
Human: Kelly (female)	Sigma Aldrich	92110411
Mouse: CT26.WT (female)	ATCC	CRL-2638
Mouse: 2208L (female)	Laboratory of Prof. Thomas Westbrook	N/A
Mouse: MMTV PyMT-M (female)	Laboratory of Prof. Thomas Westbrook	N/A
Mouse: AT3 (female)	Sigma Aldrich	SCC178
Fall armyworm (*Spodoptera frugiperda*): Sf9	ATCC	CRL-1711

**Experimental models: Organisms/strains**		

Athymic Nude-Foxn1nu mice (female)	Envigo	069

**Software and algorithms**		

GraphPad Prism	GraphPad Software	https://www.graphpad.com/scientific-software/prism/
Adobe Illustrator	Adobe	https://www.adobe.com/
FlowJo	Becton, Dickinson & Company	https://www.flowjo.com/
Schrödinger Suite v2021-2	Schrodinger	https://www.schrodinger.com/
R	The R foundation	https://www.r-project.org/
Image Studio Lite	Licor	https://www.licor.com/bio/image-studio-lite/
Proteome Discoverer 2.4	ThermoFischer Scientific	https://www.thermofisher.com/uk/en/home/industrial/mass-spectrometry/liquid-chromatography-mass-spectrometry-lc-ms/lc-ms-software/multi-omics-data-analysis/proteome-discoverer-software.html?gclid=Cj0KCQjw3a2iBhCFARIsAD4jQB2G8fbfb9HguqcFtv-W2hdY-gAsRHgJA-UY2bYiJDsgVGWx86mdrI8aAsC3EALw_wcB&cid=E.23CMD.DL103.12911.01&ef_id=Cj0KCQjw3a2iBhCFARIsAD4jQB2G8fbfb9HguqcFtv-W2hdY-gAsRHgJA-UY2bYiJDsgVGWx86mdrI8aAsC3EALw_wcB:G:s&s_kwcid=AL!3652!3!334040549172!p!!g!!proteome%20discoverer&gad=1
R Bioconductor limma package	Bioconductor	http://bioconductor.org/packages/release/bioc/html/limma.html
Bio-PDB-Structure perl package	meta:cpan (by Raul Alcantara)	https://metacpan.org/pod/Bio::PDB::Structure::Atom

### Resource availability

#### Lead contact

Further information and requests for resources and reagents should be directed to and will be fulfilled by the Lead Contact, Habib Bouguenina (habib.bouguenina@icr.ac.uk).

#### Materials availability

Plasmids and associated vector maps generated as part of this study are available upon request but may require a Materials and Transfer Agreement.

### Experimental model and study participant details

#### Bacterial strains

NEB® 5-alpha Competent *E. coli* (New England Biolabs) and BL21-AI™ One Shot™ Chemically Competent *E. coli* (ThermoFischer Scientific) were used to express all constructs used in this manuscript.

#### Cell lines

HMEC (adult female) h-TERT immortalised human mammary epithelial cells were kindly provided by Prof Martin Eilers (Würzburg university, Germany) and SUM159 (adult female) triple negative breast cancer cells were cultured in DMEM/F-12 (1:1)+ GlutaMAXTM-I (Gibco/Life Technologies), containing 10% (v/v) heat-inactivated FBS (Gibco/Life Technologies), supplemented with 15 mM HEPES (Sigma-Aldrich), 10 μg/mL insulin, human recombinant, zinc solution (Gibco/Life Technologies), 0.5 μg/mL hydrocortisone (Sigma-Aldrich) and 20 ng/mL epidermal growth factor (EGF) recombinant human protein (Life Technologies). HEK293T (embryonic female) human epithelial kidney cells, MDA-MB-231 (adult female) triple negative breast cancer cells, murine mammary cancer lines (2208L, PyMT and AT3 (female)) were cultured in DMEM (Gibco/Life Technologies), containing 25 mM D-glucose, 4 mM L-glutamine, 1 mM sodium pyruvate and supplemented with 10% (v/v) FBS and 1% nonessential amino acids (Gibco/Life Technologies). Kelly (female) human neuroblastoma cells and CT26 murine colorectal cancer cells were cultured in RPMI 1640 + 2mM Glutamine (Gibco/Life Technologies), containing 10% (v/v) heat inactivated FBS (Gibco/Life Technologies).

All cell lines were authenticated by STR profiling and controlled for mycoplasma contamination. Cultures were incubated 37°C in a humidified atmosphere with 5% CO2.

#### Animals

For the *in vivo* experiment, female 3-4 week-old Athymic Nude-Foxn1nu mice were ordered from Envigo (Order code: 069). Animals were allowed to acclimate for a minimum of 7 days prior to manipulation. Mice were housed in ventilated cages in a pathogen-free animal facility under a 14h light/10h dark cycle and had access to food and water *ad libitum* in the Baylor College of Medicine vivarium facility. Mice were handled in accordance with Baylor College of Medicine IACUC regulations and guidelines. All work was conducted under Baylor College of Medicine animal protocol AN-6672.

### Method details

#### Antibodies and cDNA plasmids

The following antibodies were used for western blotting: Anti-cMYC Y69 (Rabbit monoclonal Abcam ab32072), Anti-KRAS(G12V mutant specific D2H12) (Rabbit monclonal Cell Signaling Technologies (#14412), Anti-Phospho MEK-1 (T292) (Rabbit monoclonal Abcam ab76314),Anti-beta Actin (Mouse monoclonal abcam 8226, Mouse monoclonal Sigma Aldrich A1978), p21 (Rabbit monoclonal Cell Signaling Tech 12D1 2947S), EGFP (Rabbit polyclonal, Invitrogen A11122), Vinculin (Mouse monoclonal Sigma Aldrich, V9131), HA (Rabbit monoclonal Cell Signaling Technologies, 3724). IRDye® 680LT Goat anti-Mouse IgG (Licor, 926-68020) and IRDye® 800CW Goat anti-Rabbit IgG (Licor, 926-32211) were used as secondary antibodies.

The degron containing constructs were commercially synthetized (Invitrogen-GeneArt). Two back bones were used in this study: pLVX-puro for the doxycycline inducible systems and pHAGE-PGK DEST for the Flag-HA N terminally tagged constructs.

#### Design and selection of the DCD tags

The first group of DCD tags (DCDs1-6) were designed based on the neo-substrates GSPT1 and CK1α. Crystal structures of these neo-substrates in complex with DDB1-CRBN+IMiD are publicly available and formed the basis for our designs (Figure S2A,B).^23^^,^^47^ The DCD motifs derived from GSPT1 (DCDs1-5) all contained the glycine (G437) β−hairpin loop located within the C-terminal β−barrel domain and could be differentiated based on the presence of two C-terminal β-barrels of GSPT1 (DCD1), the last C-terminal β-barrel only (DCD2), the minimal glycine β−hairpin loop only (DCD3) or the β−hairpin loop extended with truncated aa stretches from the C terminal β-barrel (DCD4,5) (Figure S1). The CK1α-based DCD6 was restricted to the Glycine (G40) hairpin loop (Figure S1).

The second group of DCD tags (DCDs7-20) was designed based on the C2H2 Zinc Finger neo-substrates (Ikaros, Aiolos, Eos and ZFP91). The published crystal structures suggest that C2H2 Zinc finger domains comprise an N-terminal β-hairpin and a C-terminal α-helix coordinated by multivalent zinc molecules, with a conserved binding mode to CRBN (Figure S2C). Ikaros, Aiolos, Eos and ZFP91 all contain many C2H2 ZF domains, several of which bearing the CxxCG signature of CRBN ZF neo-substrates, *in vitro* binding, cellular degradation assays and docking studies showed that the critical glycine β-hairpin loop involved in the binding to CRBN was located in Ikaros ZF2 aa145-167, Aiolos ZF2 aa146-168, Eos ZF2 aa187-209 and ZFP91 ZF4 aa400-422.^20^^,^^24^^,^^25^ These sequences were selected for designing DCDs7-16, and 18–20. To identify the optimal sequence for protein degradation, the sequences of these DCDs were extended to include multiple combination of adjacent ZF domains. We also tested Aiolos ZF5-ZF6 aa 448–509 (DCD17) to confirm the importance of ZF2 in Aiolos for inducing degradation.

The final group, DCDs 21–23, was based on a degron hybrid between two ZF CRBN substrates: Ikaros and ZFP91. The work of Sievers et al. previously demonstrated that chimeric Aiolos/ZFP91 ZF had higher affinity for CRBN compared to the Aiolos or ZFP91 ZFs^25^ and that a stretch of three consecutive arginine residues within Ikaros ZF3 (R183-R185) were critical in enhancing the binding to CRBN. Based on these data, and our own data from the second group of DCDs showing strong degradation using Ikaros-based DCD19 (Figures S3 and S4), we generated ZFP91/Ikaros chimeras by fusing the β-hairpin of ZFP91 ZF4 (aa400-410) which contains the apical Glycine to the α-helix of Ikaros ZF2 (aa156-167), followed by various sized extensions from Ikaros sequence, all maintaining the critical arginine stretch of Ikaros ZF3 (R183-R185)^25^ (Figures S1–S4).

The sequence of DCD23, which was selected as the optimal sequence for iTAG was designed as follows: Ikaros ZF1(α-helix)(aa129-144); ZFP91 ZF4(β-haipin containing the apical Glycine) (aa400-410); Ikaros ZF2(α-helix)-ZF3(β-hairpin) (aa156-188).

#### Virus production and cell line transduction

For the pLVX-puro doxycycline inducible constructs: viral particle production, HEK293T cells were seeded prior to transfection at a density of 80–90%. Cells were transfected with the lentiviral vector plasmid, psPAX2 and pMD.2G using Lipofectamine 3000™ (ThermoFischer) and following manufacturer’s recommendations. Viral particle titer was determined using the Lenti-XTM GoStixTM kit (Takara Bio), according to manufacturer’s instructions. Virus-containing supernatants were harvested 48 and 72 h post-transfection, filtered through a 0.45 μm filter (Sartorius Stedim) and stored at −80°C.

For cell transduction, HMEC cells seeded 2 days before were infected at an MOI of 5 by adding the virus-containing supernatants with polybrene at 4 μg/mL. The plates with the infected cells were centrifuged at 900 g, 32°C for 90 min and cultured for 2 passages before inducing constructs expression with 1 μg/mL of doxycycline for 24h and isolating EGFP positive cells using Fluorescence-activated cell sorting (FACS).

For the pHAGE-PGK constructs: DCD23, SuperDegron and FKBP12^F36V^ were separately cloned into a pHAGE-PGK DEST backbone. cDNA encoding the indicated protein was then cloned from pENTR221 into the pHAGE-PGK-DCD23, pHAGE-PGK-SuperDegron or pHAGE-PGK-FKBPF36V DEST vector using Gateway cloning. SUM159 cells were transduced with the indicated lentivirus and selected with puromycin.

#### Evaluating iTAG degradation with flow cytometry

Cells stably transduced with the iTAG tagged constructs were treated with 1 μg/mL of doxycycline for 24h to induce constructs expression. Subsequently, cells were exposed to IMiD/CELMoD degrader compounds (CC885, lenalidomide, pomalidomide, CC220, and CC122) at 10μM for 4h to capture acute degradation events. Following degrader treatment cells were collected and DAPI was added to the cell suspension at 1 μg/mL to allow the exclusion of dead cells. EGFP signal was measured using BD LSR II flow cytometer (BD Biosciences). FlowJo 10 Software (FlowJo) was used for data processing.

#### Cell lysis, protein electrophoresis and western blotting

Cells were lysed with SDS lysis buffer (2% SDS, 10% Glycerol, 50mM Tris pH 6.8) and boiled at 95°C for 30min. Protein concentration was measured using Pierce BCA Protein Assay Kit (ThermoFischer) following manufacturer’s recommendations. Samples were ran in NuPAGETM 10% Bis-Tris Midi electrophoresis gels (Invitrogen) using NuPAGETM MES SDS running buffer (ThermoFischer Scientific). Proteins were subsequently transferred to an Immobilon-P polyvinylidene Difluoride (PVDF) membrane (Merck Millipore) using the wet-transfer method (Bio-Rad) in a transfer buffer consisting of 20% (v/v) methanol and 10% protein electrophoresis buffer (24.77 mM Tris and 0.192 M glycine).

For western blotting membranes were blocked in a solution of 5% (w/v) bovine serum albumin (BSA; Sigma-Aldrich) dissolved in TBS-0.1% Tween 20 (Sigma-Aldrich) then incubated at 4°C overnight with the primary antibodies diluted in 5% (w/v) BSA/TBS-T. The membranes where then washed and incubated with anti-mouse and/or anti-rabbit secondary antibodies (LI-COR Biosciences) for 2h at RT diluted in 5% (w/v) BSA/TBS-T then washed and imaged using the LI-COR Odyssey (LI-COR Biosciences). Images were analyzed using Image Studio Lite software (LI-COR Biosciences).

#### Cell viability assay

HMEC, HEK293T, Kelly and MDA-MB-231 cells were seeded in 96-well plate in triplicates and treated with CC885, Thalidomide, Lenalidomide, Pomalidomide, CC220 or CC-122 for 5 days. By endpoint, CellTiter-Blue reagent (Promega) was added to each well, incubated for 3 h at 37°C and absorbance was measured with a plate reader at 570nm. Average background absorbance value was subtracted from all readings when plotting the viability curve.

#### Recombinant proteins production and purification

##### Human CRBN/DDB1 complex

Full length human 6His-ZZ-CRBN and DDB1-Strep proteins were co-expressed in sf9 insect cells. Baculoviruses were generated using the Bac-to-Bac - Baculovirus Expression System (Thermo Fisher Scientific A11101). Sf9 cells growing at 27°C in shaker flasks with Sf-900™ III SFM media were infected with 10–30 μL virus/10^7^ cells and harvested 48 to 72 h post infection. Cell pellets were stored at −80°C. Cells were thawed and re-suspended with a 4 to 5-fold volume of IMAC buffer (50 mM Tris pH8.0, 500 mM NaCl, 0.5 mM TCEP, 5% glycerol) complemented with 1 mM MgCl_2_, 1x cOmplete ULTRA protease inhibitors and 12.5 U/mL Benzonaze. Cells were lysed by sonication followed by centrifugation (55,900 g, 45 min, 4°C) and filtration (1.2 μm syringe filter). Clarified lysate was applied to 2 × 5mL HisTrap FF columns, washed with IMAC buffer with 20 mM imidazole and eluted with IMAC buffer with 250 mM imidazole. IMAC elute was applied onto a Superdex 200 column (HR26/60). The peak elutes were pooled, diluted to 50 mM NaCl and loaded onto a 6 mL Resource Q column. Following a 50 to 500 mM NaCl gradient elution, peak fractions were pooled and loaded back onto a Superdex 200 column (HR26/60) in a buffer containing 50 mM HEPES pH 8.0, 200 mM NaCl, 0.5 mM TCEP and 5% glycerol. Peak fractions were pooled, concentrated and stored at −80°C.

##### DCD23

The sequence coding for a slightly shorter version of DCD23 than used in cells, consisting of residues 141 to 144 of Ikaros followed by residues 400–410 of Zfp91 and residues 156–188 of Ikaros, was codon optimized for production in *E. coli* and synthesised by Eurofins. It was subcloned into pET48b plasmid between XmaI and XhoI restriction sites to generate a plasmid coding for a Trx-6His-HRV3C-DCD23 protein. Transformed BL21-AI *E. coli* cells were grown in TB media supplemented with 50 mg/L Kanamycin at 37°C until an OD_600 nm_ of 0.6 was reached. Protein expression was then induced by addition of 0.2 mM IPTG and 0.2% Arabinose. Expression was carried out at 18°C for 18 h. Cells were harvested by centrifugation at 5500*g* for 30 min at 4°C, and stored at −80°C. Cells were re-suspended in a buffer composed of 50 mM HEPES pH 7.4, 200 mM NaCl, 1 mM MgCl2, 0.25 mM TCEP, 1x complete ULTRA protease inhibitors, 1 mg/mL Lysozyme and 12.5 U/mL Benzonaze. Cells were lysed by sonication followed by centrifugation at 56,000 g for 20 min at 4°C. The supernatant was loaded onto a HisTrap FF column, and eluted with 250 mM Imidazole. The Trx-6His-HRV3C tag was cleaved with HRV-3C protease, and DCD23 further purified with a Superdex75 16/60 followed by reverse-HisTrap FF and MonoS HR5/5 steps. Finally, the resulting DCD23 was ran through a PD10 column to exchange buffer to 50 mM Tris pH 8, 200 mM NaCl, 0.25 mM TCEP, and stored at −80°C.

#### IMiD-based fluorescence polarisation assay

All FP assays were run in Black 384-well ProxiPlate Plus (Perkin-Elmer, USA), in a buffer containing 20 mM HEPES pH 8.0, 150 mM NaCl, 0.5 mM TCEP and 0.05% Tween 20, with a final assay volume of 10 μL. An Echo E5XX (Beckman Coulter, USA) acoustic liquid dispenser was used to create final concentration ranges of compound from 300 μM to 0.9375 nM, with a final DMSO concentration of 3%. WT full length CRBN/DDB1 complex was added with a Tempest liquid handler (Formulatrix, USA) to all wells to a final concentration of 80 nM, except for low controls where WT CRBN was substituted with mutant Y384A-W386A CRBN (mutant unable to bind the probe). The Echo was also used to add 5 nM final concentration of Sulfo-Cy5 fluorescent IMiD-based probe (Figure S6A) to each well. Plates were sealed, centrifuged at 200 g for 1 min and stored overnight at 4°C. Fluorescence Polarization and total fluorescence were read using a PHERAstar FSX plate reader (BMG Labtech, Germany).

The Kd determination was carried out in a 384 well black Proxiplate (Perkin-Elmer, USA). WT hCRBN was dispensed using an Echo acoustic liquid dispenser (Beckman, USA) to final concentrations of 5.9 μM–9.7 nM. IMiDs were added to a final concentration of 10 μM except CC-885 which was 1 μM final concentration due to solubility issues. Cy5 labeled Aiolos & Zn(CH3COO)2 were added to a final concentration of 10 nM. After overnight incubation, fluorescence polarization was measured using a Pherastar plate reader (BMG Labtech, Germany). Kd values for Cy5- Aiolos to CRBN binding were calculated for each IMiD using Prism software.

#### Aiolos peptide-based TR-FRET assay

All TR-FRET assays were ran in Black 384-well ProxiPlate Plus (Perkin-Elmer, USA), in a buffer containing 20 mM HEPES pH 8.0, 150 mM NaCl, 0.5 mM TCEP and 0.05% Tween 20, with a final assay volume of 10 μL. An Echo E5XX (Beckman Coulter, USA) acoustic liquid dispenser was used to create final concentration ranges from 90 μM down to 2.25 nM for DCD23. Final concentrations to 5 nM WT full length CRBN/DDB1 complex, 750 nM Sulfo-Cy5 fluorescent Aiolos-based peptidic probe (Figure S6C) (Cambridge Research Biochemicals, UK) and 0.5 nM MAb Anti-6HIS-Terbium cryptate Gold (Cisbio, France) were added with a Tempest liquid handler (Formulatrix, USA). Finally, degraders were added to a final concentration of 10 μM (or 1 μM for CC885 due to solubility issues) with the Echo. Plates were sealed, centrifuged at 200 g for 1 min and stored overnight at 4°C. The TR-FRET signals were read using a PHERAstar FSX plate reader (BMG Labtech, Germany). Final signal was measured as the ratio:

$$TR-FRET\;signal=\frac{chanel1\;\left(665nm\right)}{chanel2\;\left(620nm\right)}$$

Chanel1 at 665nm representing a positive FRET and chanel2 at 620nm representing the emission of the terbium tag when no FRET occurs (Figure S6C).

Kd values were used along with the TR-FRET IC_50_ values to calculate the Ki using the Cheng-Prusoff equation:

Ki=IC50/[1+(HTRFlabelledligandconcentration)/Kd]

#### *In vivo* analysis of degradation

MDA-MB-231 cells (5 × 10^6^ cells) engineered to express FLuc-EGFP-DCD23 or FLuc-EGFP(P2A)DCD23 were transplanted subcutaneously into the right flank of 4–5 week old Athymic Nude-Foxn1nu (Envigo) mice. Subcutaneous tumor volume was measured using calipers. Animals were administered sucrose water with doxycycline (2 mg/mL) for 3–5 days when tumors reached 200-350mm^3^. After doxycycline administration, animals were randomized for vehicle or CC220 (30 mg/kg in 0.5% carboxymethyl cellulose (Na salt) with 0.25% Tween80 in water) treatment. Tumor luminescence was quantified using non-invasive bioluminescence imaging, with luminescence measured 15s post retro-orbital luciferin injection (150 mg/kg) using an IVIS imager. Tumor luminescence was measured before and at the indicated timepoint after vehicle or CC220 administration. Tumors were then harvested, and flash frozen for further analysis.

#### Mass spectrometry–based proteomics

##### Sample preparation

Samples for quantitative proteomics were prepared using the SimPLIT workflow.^60^ Briefly, the cell pellets were lysed in a buffer containing 150 μL 0.1 M triethylammonium bicarbonate (TEAB), 1% sodium deoxycholate (SDC), 10% isopropanol, 50mM NaCl, 5 mM tris-2-carboxyethyl phosphine (TCEP), 10 mM iodoacetamide (IAA), universal nuclease, and supplemented with protease and phosphatase inhibitors. Samples were bath sonicated for 5 min, incubated at RT for 45 min and protein concentration was measured with the Bradford Protein Assay (Biorad) according to manufacturer instructions. Aliquots containing 30 μg of total protein were used for protein digestion. The resultant peptides were labeled with a tandem mass tag (TMTpro) multiplex reagent vial (Thermo Scientific) according to the manufacturer instructions. All the TMT-labelled samples were combined in equal amounts to a single tube. The TMT-peptide mixture was acidified with 1% formic acid and the precipitated SDC was removed by centrifugation. The sample was then dried with a centrifugal vacuum concentrator.

##### High-pH reversed-phase peptide fractionation

Offline peptide fractionation was based on high pH Reverse Phase (RP) chromatography using the Waters XBridge C18 column (2.1 × 150 mm, 3.5 μm) on a Dionex Ultimate 3000 HPLC system at a 0.85% gradient with flow rate 0.2 mL/min. Mobile phase A was 0.1% ammonium hydroxide, and mobile phase B was 100% acetonitrile, 0.1% ammonium hydroxide. Retention time-based fractions are collected and pooled into thirty samples for LC-MS analysis.

##### Liquid chromatography-mass spectrometry analysis

LC-MS analysis was performed on the Dionex Ultimate 3000 UHPLC system coupled with the Orbitrap Lumos mass spectrometer (Thermo Scientific). Samples were analyzed with the EASY-Spray C18 capillary column (75 μm × 50 cm, 2 μm) at 50°C. Mobile phase A was 0.1% formic acid and mobile phase B was 80% acetonitrile, 0.1% formic acid. The gradient separation method was as follows: 90 min gradient up to 38% B, for 10 min up to 95% B, for 10 min isocratic at 95% B, re-equilibration to 5% B in 5 min, for 10 min isocratic at 5% B. For MS analysis, precursors between 375 and 1,500 *m*/*z* were selected, with mass resolution of 120,000, automatic gain control (AGC) of 4 × 105, and IT (injection time) of 50 ms, with the top speed mode in 3 s, and the precursors were isolated for collision-induced dissociation (CID) fragmentation with a quadrupole isolation width of 0.7 Th (Thomson unit). Collision energy was set at 35%, with AGC at 1 × 104 and IT at 50 ms. MS3 quantification was obtained with higher-energy collisional dissociation (HCD) fragmentation of the top 5 most abundant CID fragments isolated with synchronous precursor selection (SPS). Quadrupole isolation width was set at 0.7 Th, collision energy was applied at 65%, and the AGC setting was at 1 × 105 with IT at 105 ms. The HCD MS3 spectra were acquired for the mass range 100–500 with a resolution of 50,000. Targeted precursors were dynamically excluded for further isolation and activation for 45 s with 7 ppm mass tolerance.

##### Database search and protein quantification

The SEQUEST-HT search engine was used to analyze the acquired mass spectra in Proteome Discoverer 2.4 (Thermo Scientific) for protein identification and quantification. The precursor mass tolerance was set at 20 ppm, and the fragment ion mass tolerance was set at 0.5 Da. Spectra were searched for fully tryptic peptides with maximum 2 mis-cleavages. TMTpro on lysine residues and peptide N termini (304.2071 Da) and carbamidomethylation of cysteine residues (+57.0215 Da) were set as static modifications while oxidation of methionine residues (+15.9949 Da) and deamidation of asparagine and glutamine (+0.9848 Da) was set as a variable modification. Peptide confidence was estimated with the Percolator. The peptide FDR was set at 0.01, and validation was based on the q value and a decoy database search. All spectra were searched against UniProt-SwissProt proteomes of *Homo sapiens* protein entries appended with contaminants. The reporter ion quantifier node included a TMTpro quantification method with an integration window tolerance of 15 ppm and integration method based on the most confident centroid peak at the MS3 level. Only unique peptides were used for quantification, with protein groups considered for peptide uniqueness. Peptides with an average reporter signal-to-noise ratio of >3 were used for protein quantification. Correction for isotopic impurity of reporter quantification values is applied. Only spectra with at least 50% of the SPS masses matching to the identified peptides are used for quantification.

#### Proteomics hit discovery

Peptide abundance data were initially filtered to removed entries mapping to a panel of 193 known contaminant proteins across the human, bovine and mouse proteomes (e.g. keratins, trypsin, thrombin, albumin) or flagged as such in the Peptide abundance input file. Peptides with missing abundance information for >30% of the TMT channels were excluded from downstream analysis. Any remaining missing abundance values were imputed to the lowest observed abundance per peptide. To correct for experimental bias, normalisation using peptide amount per channel was performed.

Normalised peptide abundances for unique peptides identified with high confidence were summed by protein group and log2 transformed to generate normalised protein abundance values. Protein ratios for desired comparisons were computed using the moderated t-test functionality in the R Bioconductor limma package.^61^

#### PDE6D Gly-loop degron discovery

The PDB experimental structure of PDE6D was scanned for close structural homologues to the CK1alpha degron G-loop (defined in PDB structure 5FQD, chain C, sequence INITNG40E). A structural heptamer was constructed for each glycine residue of the interrogated PDB chain, extending five N-terminal and one C-terminal residues to that glycine residue (the degron anchor residue). Heptamers with fully defined 3D coordinates were assessed for accessibility (minimum degron anchor residue accessibility 0.3 (Angstrom2), minimum heptamer residue accessibility 0.35 (Angstrom2), with at least 4 heptamer residues passing accessibility thresholds), presence of a backbone-to-backbone hydrogen bond between residues at position +1 and −4 relative to the degron anchor Gly, and low helical content (two or less heptamer residues). The above parameters were computed using DSSP^62^^,^^63^ on individual PDB chains.

Qualifying heptamers were superposed to the CK1alpha degron G-loop heptamer and the alpha carbon RMSD was computed using the Bio-PDB-Structure perl package (https://metacpan.org/dist/Bio-PDB-Structure). The PDB chain structure of heptamers with RMSD values below 2 Angstrom was further evaluated for structural clashes against CRBN (PDB structure 5FQD, chain B, including the Lenalidomine ligand LVY) using the Bio-PDB-Structure perl package. Side chains were excluded from the clash computation. Clashes were classed as Lenalidomide, degron, near-degron (within 5A of the heptamer) and distal (beyond 5A of the heptamer). Distal clashes were ignored. Successful degron G-loops exhibited no Lenalidomide, degron or near-degron clashes.

#### In silico docking and molecular dynamics

A chimera homology model was generated within the Schrödinger Suite v2021-2 for mouse CRBN (UniProt ID: Q8C7D2) using two structural templates as sorted by resolution across the set available for the corresponding species: a) the thalidomide binding domain of mouse CRBN (PDB ID: 5YJ0); b) the human CRBN structure in complex with DDB1 and CK1α (PDB ID: 5FQD, 97% sequence identity to mouse CRBN) to fill in sensor loop residues (S341-E358 in 5FQD), Lon and HB domains missing from the first template. All protein structures were prepared using the Protein Preparation Wizard in Maestro.^64^ The homology model was refined through 2500 MacroModel minimisation iterations using the Polak-Ribier Conjugate Gradient (PRCG) method, the OPLS4 force-field^65^ and an implicit water solvation model. The structure of FPFT-2216 was prepared using LigPrep to generate tautomers, stereoisomers and protonation states at pH 7.0 ± 2.0 as default. Docking of FPFT-2216 against the minimised homology model of mouse CRBN was carried out with Glide SP^66^^,^^67^ in a receptor grid centered at the centroid of Trp383, Trp389 and Trp403, namely in the tri-Trp pocket. The complex formed by the best scoring ligand pose was refined through a molecular dynamics simulation using the Desmond package.^68^ The system was neutralised, immersed in a TIP3P water box of orthorhombic shape extended up to 10 Å in each direction from system atoms and prepared under periodic boundary conditions with the OPLS4 force-field using the System Builder in Desmond. Following an initial relaxation with the default protocol, a 100 ns MD simulation (1000 frames) was carried out on the solvated complex in the NPT ensemble at 1.01325 bar and 300 K. Representative conformations were obtained by clustering MD trajectory frames on backbone atoms of binding site residues with the Desmond Trajectory Clustering. Protein-protein docking simulations were performed with Piper^69^ using the refined CRBN:FPFT-2216 model as receptor and the PDE6D apo structure as ligand (PDB ID: 5T4X). The ternary complex pose selected from protein-protein docking was subjected to three independent MD replicas with randomised initial velocities using the same parameters as described above. A total of 3000 MD trajectory frames were analyzed to extract interatomic distances using the Schrödinger Python APIs (www.schrodinger.com/pythonapi).

### Quantification and statistical analysis

Flow cytometry data were transferred to GraphPad Prism for analysis and presentation. Number of experiments and analysis are indicated in the figure legend.

For the proteomics data, normalised peptide abundances for unique peptides identified with high confidence were summed by protein group and log2 transformed to generate normalised protein abundance values. Protein ratios for desired comparisons were computed using the moderated t-test functionality in the R Bioconductor limma package.^61^

## Data Availability

Proteomic data has been uploaded to the PRIDE data repository, under the project accession code PXD038299. This study did not generate new code. Any additional information required to reanalyse the data reported in this paper is available from the lead contact upon request.
