# iTAG an optimized IMiD-induced degron for targeted protein degradation in human and murine cells

**DOI:** 10.1016/j.isci.2024.109727

**Published:** 2024-04-14

**Authors:** Habib Bouguenina, Stephanos Nicolaou, Yann-Vaï Le Bihan, Elizabeth A. Bowling, Cheyenne Calderon, John J. Caldwell, Brinley Harrington, Angela Hayes, P. Craig McAndrew, Costas Mitsopoulos, Fernando Jr. Sialana, Andrea Scarpino, Mark Stubbs, Arjun Thapaliya, Siddhartha Tyagi, Hannah Z. Wang, Francesca Wood, Rosemary Burke, Florence Raynaud, Jyoti Choudhary, Rob L.M. van Montfort, Amine Sadok, Thomas F. Westbrook, Ian Collins, Rajesh Chopra

## Main text

(iScience *26*, 107059; July 21, 2023)

In the originally published version of this article, an error occurred in depicting the molecular mass of DCD19, DCD20, and DCD21 and the molecular mass plus sequence of DCD23 in Figures S1, S4, and S12. This error has now been corrected in the article online. The corrections do not affect the validity of the data and the conclusion. The authors apologize for any confusion this error may have caused.

**Figure:**
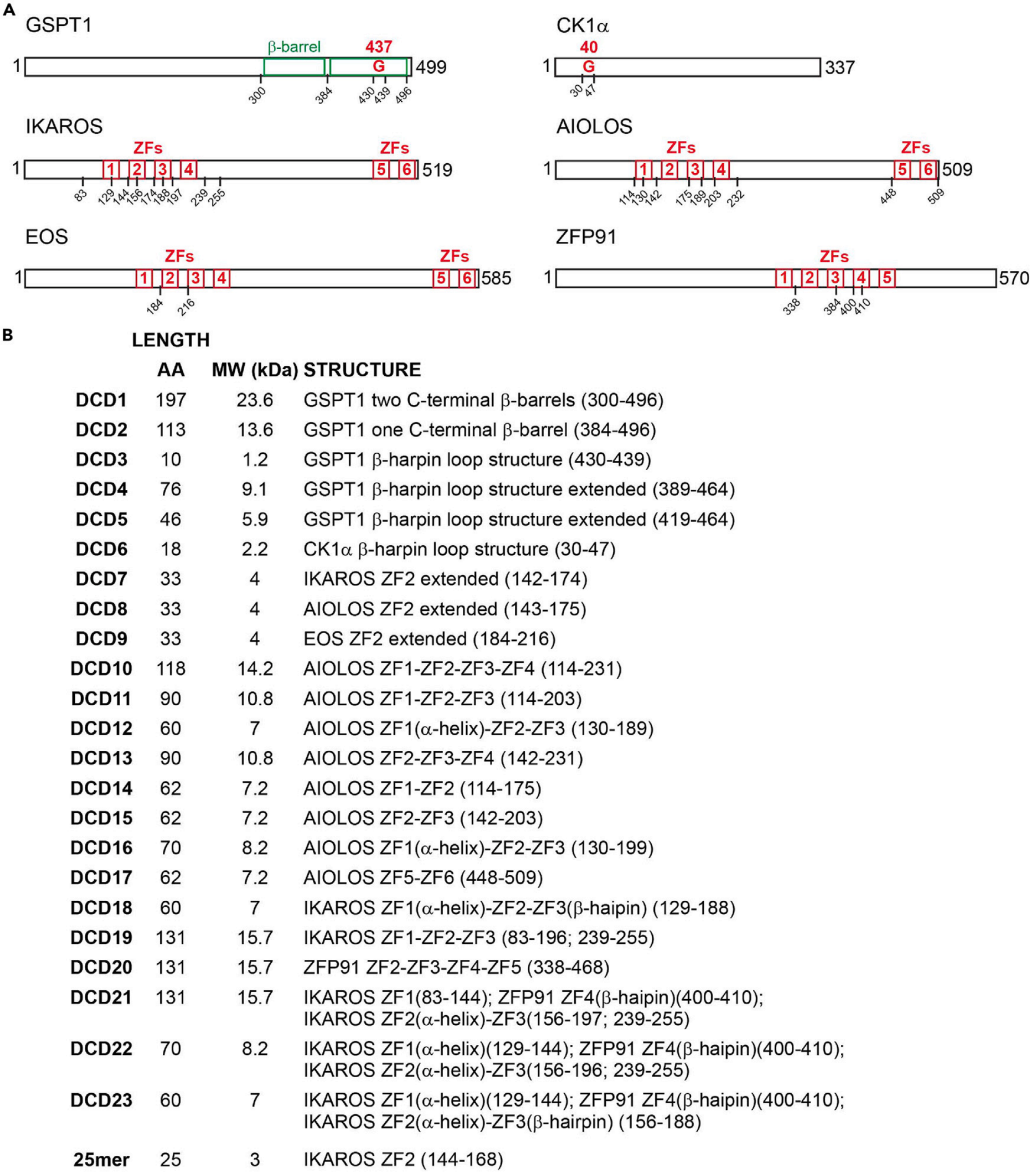
Figure S1. Representation of the Degron Containing Domains (DCDs), related to Figure 1 (original)

**Figure:**
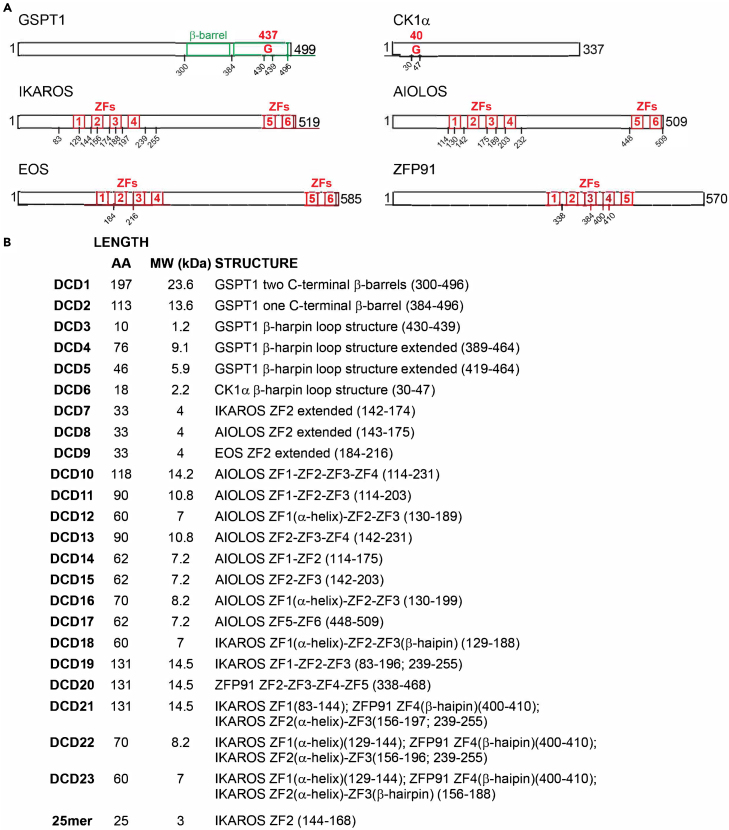
Figure S1. Representation of the Degron Containing Domains (DCDs), related to Figure 1 (corrected)

**Figure:**
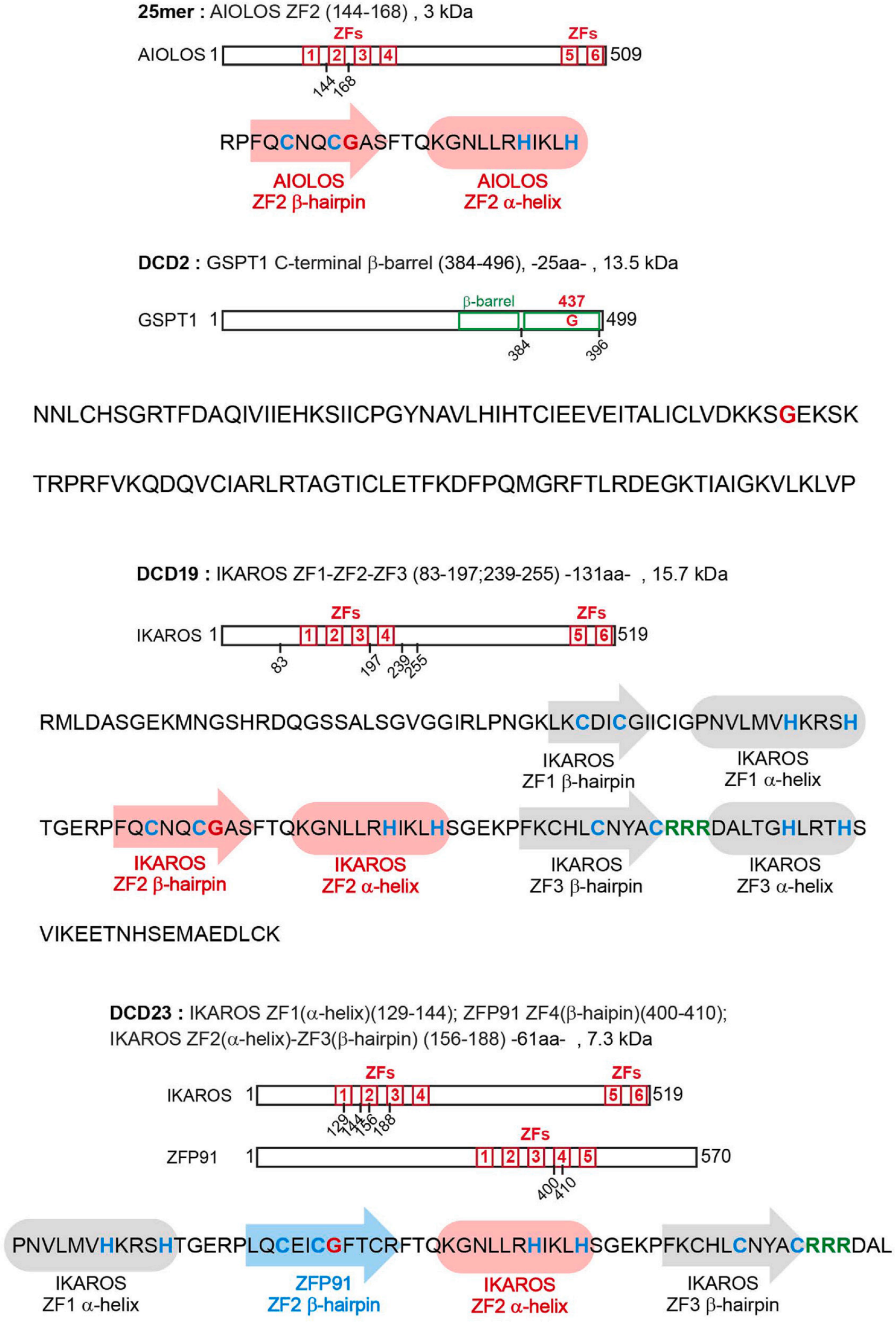
Figure S4. Sequence of Aiolos 25-mer, DCD2, DCD19, DCD23 showing the different critical motifs, related to Figure 1 (original)

**Figure:**
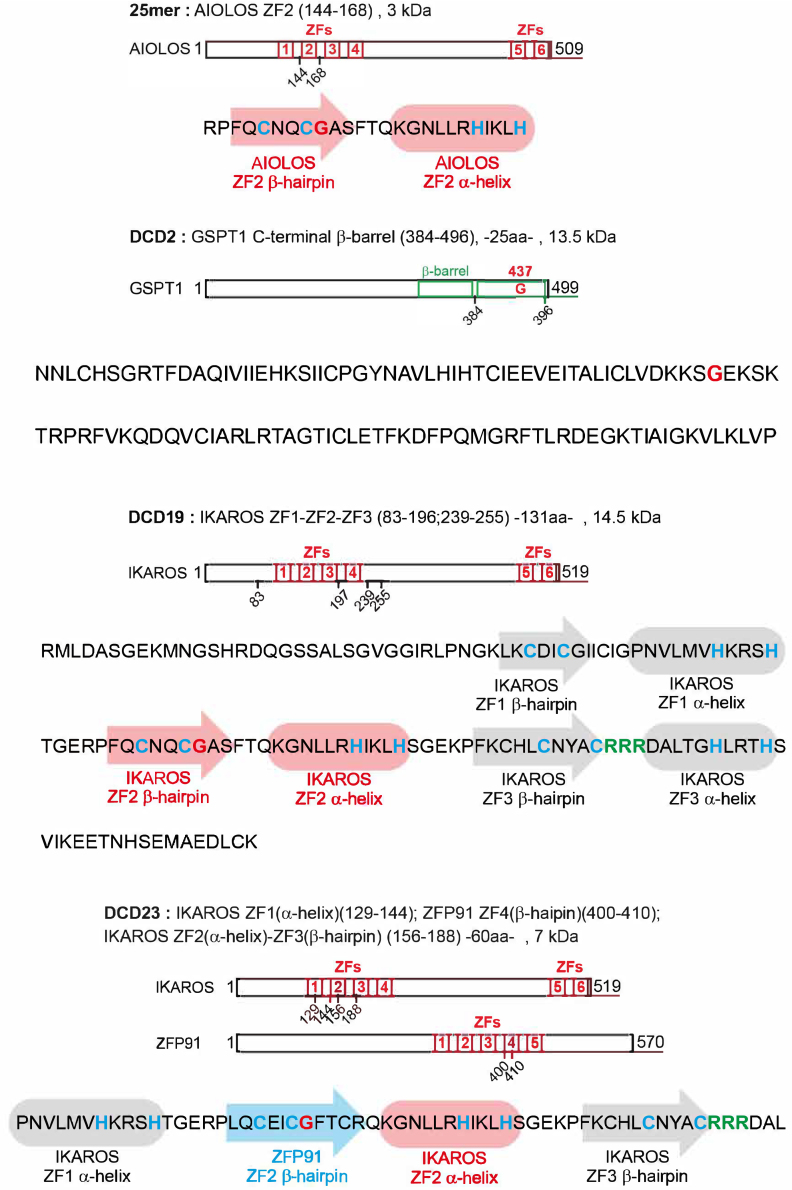
Figure S4. Sequence of Aiolos 25-mer, DCD2, DCD19, DCD23 showing the different critical motifs, related to Figure 1 (corrected)

**Figure:**
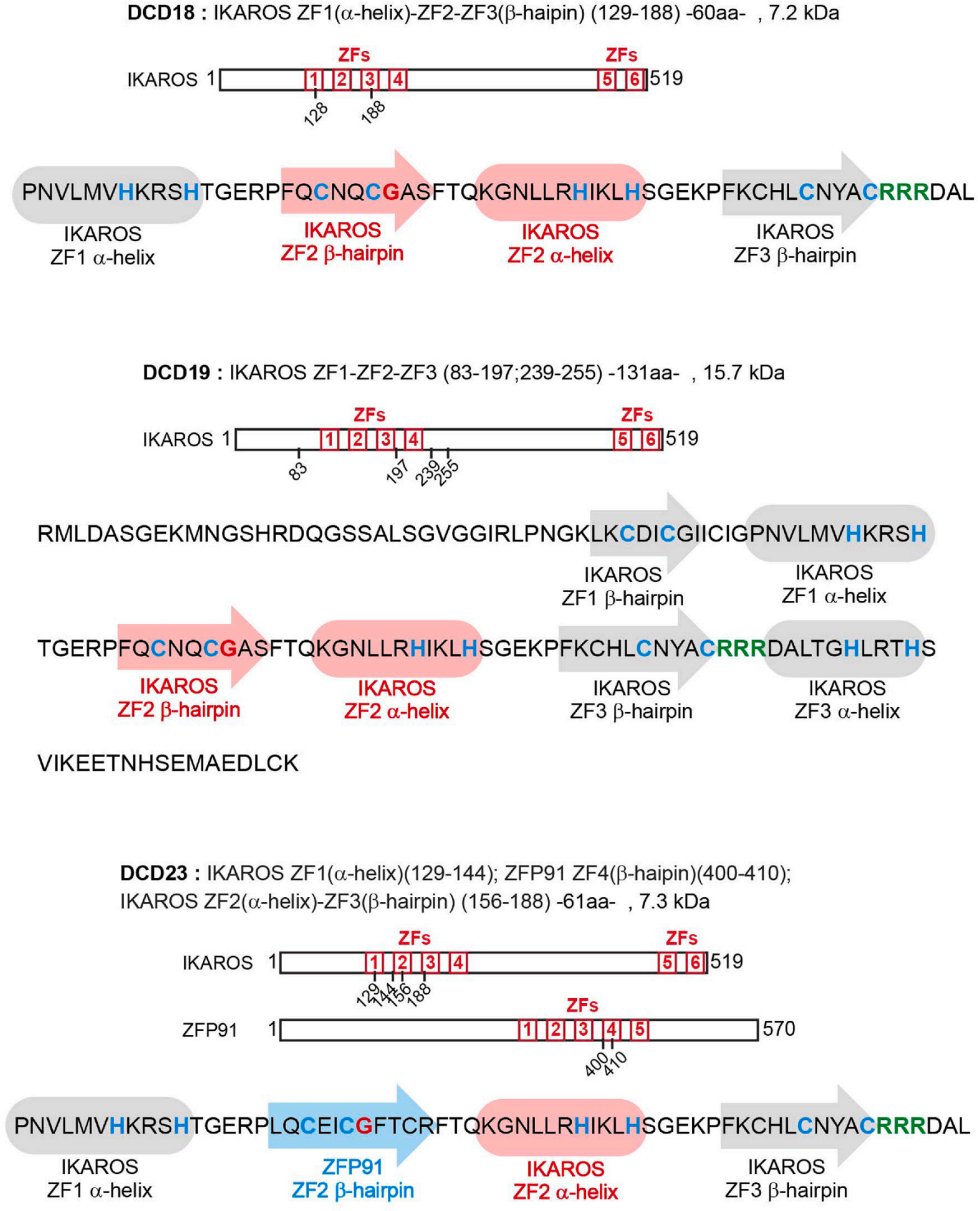
Figure S12. Sequence of DCD18, DCD19, DCD23 showing the different zinc finger motifs, related to Discussion (original)

**Figure:**
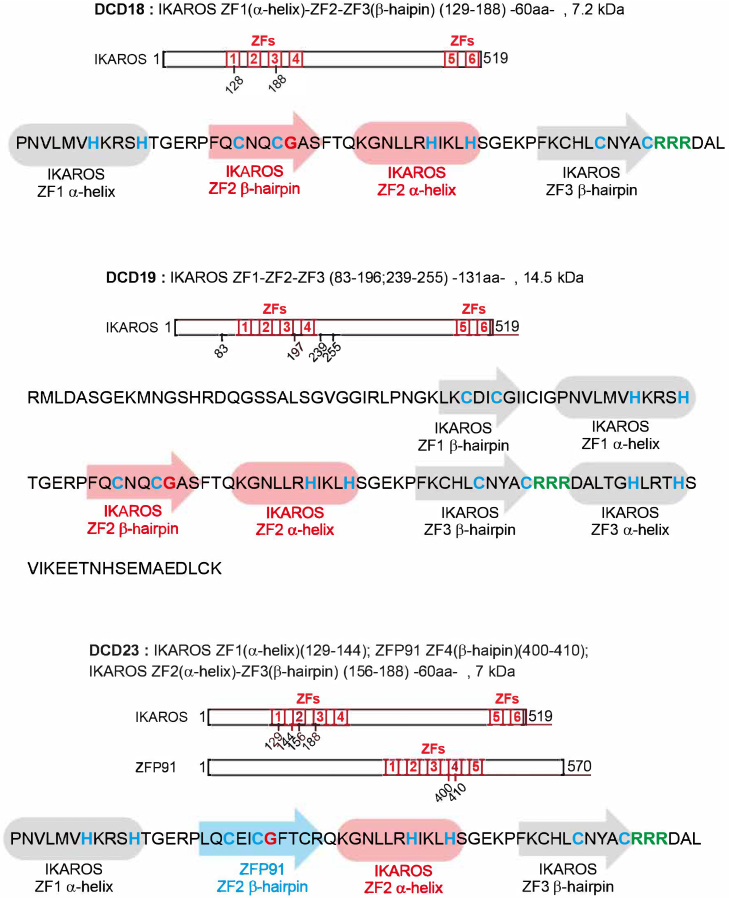
Figure S12. Sequence of DCD18, DCD19, DCD23 showing the different zinc finger motifs, related to Discussion (corrected)

