# Supplementary material for: iTAG an optimized IMiD-induced degron for targeted protein degradation in human and murine cells
Source: iScience. 2023 Jun 7;26(7):107059. doi: 10.1016/j.isci.2023.107059 (PMC10285648; doi:10.1016/j.isci.2023.107059)
Supplement: Document S1. Figures S1–S12 and Tables S1 and S2 [file mmc1.pdf]

iScience, Volume *26*

Supplemental information

iTAG an optimized IMiD-induced degron for targeted protein degradation in human and murine cells

Habib Bouguenina, Stephanos Nicolaou, Yann-Vaï Le Bihan, Elizabeth A. Bowling, Cheyenne Calderon, John J. Caldwell, Brinley Harrington, Angela Hayes, P. Craig McAndrew, Costas Mitsopoulos, Fernando Jr. Sialana, Andrea Scarpino, Mark Stubbs, Arjun Thapaliya, Siddhartha Tyagi, Hannah Z. Wang, Francesca Wood, Rosemary Burke, Florence Raynaud, Jyoti Choudhary, Rob L.M. van Montfort, Amine Sadok, Thomas F. Westbrook, Ian Collins, and Rajesh Chopra

A

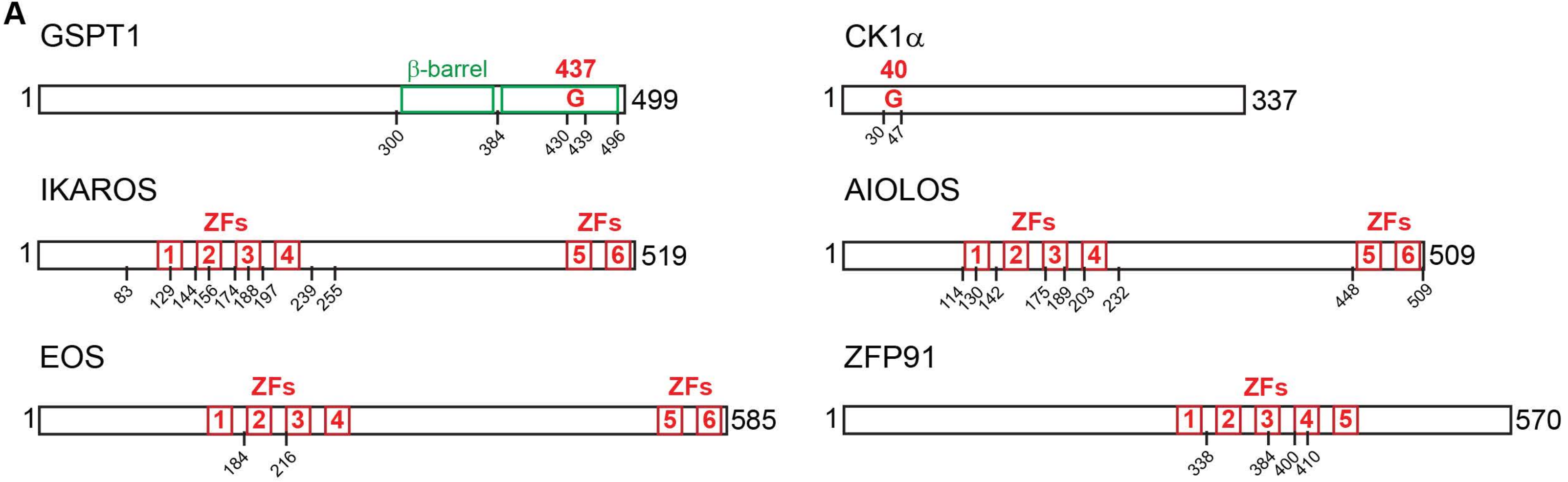

B

	LENGTH AA	MW (kDa)	STRUCTURE
**DCD1**	197	23.6	GSPT1 two C-terminal β-barrels (300-496)
**DCD2**	113	13.6	GSPT1 one C-terminal β-barrel (384-496)
**DCD3**	10	1.2	GSPT1 β-harpin loop structure (430-439)
**DCD4**	76	9.1	GSPT1 β-harpin loop structure extended (389-464)
**DCD5**	46	5.9	GSPT1 β-harpin loop structure extended (419-464)
**DCD6**	18	2.2	CK1α β-harpin loop structure (30-47)
**DCD7**	33	4	IKAROS ZF2 extended (142-174)
**DCD8**	33	4	AIOLOS ZF2 extended (143-175)
**DCD9**	33	4	EOS ZF2 extended (184-216)
**DCD10**	118	14.2	AIOLOS ZF1-ZF2-ZF3-ZF4 (114-231)
**DCD11**	90	10.8	AIOLOS ZF1-ZF2-ZF3 (114-203)
**DCD12**	60	7	AIOLOS ZF1(α-helix)-ZF2-ZF3 (130-189)
**DCD13**	90	10.8	AIOLOS ZF2-ZF3-ZF4 (142-231)
**DCD14**	62	7.2	AIOLOS ZF1-ZF2 (114-175)
**DCD15**	62	7.2	AIOLOS ZF2-ZF3 (142-203)
**DCD16**	70	8.2	AIOLOS ZF1(α-helix)-ZF2-ZF3 (130-199)
**DCD17**	62	7.2	AIOLOS ZF5-ZF6 (448-509)
**DCD18**	60	7	IKAROS ZF1(α-helix)-ZF2-ZF3(β-haipin) (129-188)
**DCD19**	131	15.7	IKAROS ZF1-ZF2-ZF3 (83-196; 239-255)
**DCD20**	131	15.7	ZFP91 ZF2-ZF3-ZF4-ZF5 (338-468)
**DCD21**	131	15.7	IKAROS ZF1(83-144); ZFP91 ZF4(β-haipin)(400-410);<br>IKAROS ZF2(α-helix)-ZF3(156-197; 239-255)
**DCD22**	70	8.2	IKAROS ZF1(α-helix)(129-144); ZFP91 ZF4(β-haipin)(400-410);<br>IKAROS ZF2(α-helix)-ZF3(156-196; 239-255)
**DCD23**	60	7	IKAROS ZF1(α-helix)(129-144); ZFP91 ZF4(β-haipin)(400-410);<br>IKAROS ZF2(α-helix)-ZF3(β-hairpin) (156-188)
**25mer**	25	3	IKAROS ZF2 (144-168)

Figure S1. Representation of the Degron Containing Domains (DCDs), related to Figure1

(A) Representation of GSPT1, CK1α, Ikaros, Aiolos, Eos and ZFP91. In each protein, the Glycine at the apex of the β-hairpin loop implicated in the binding to CRBN in GSPT1 and CK1α and the different zinc finger domains is highlighted in red.

(B) Summary of the different DCD evaluated in this study. Sequence references were obtained from UniProt data base.

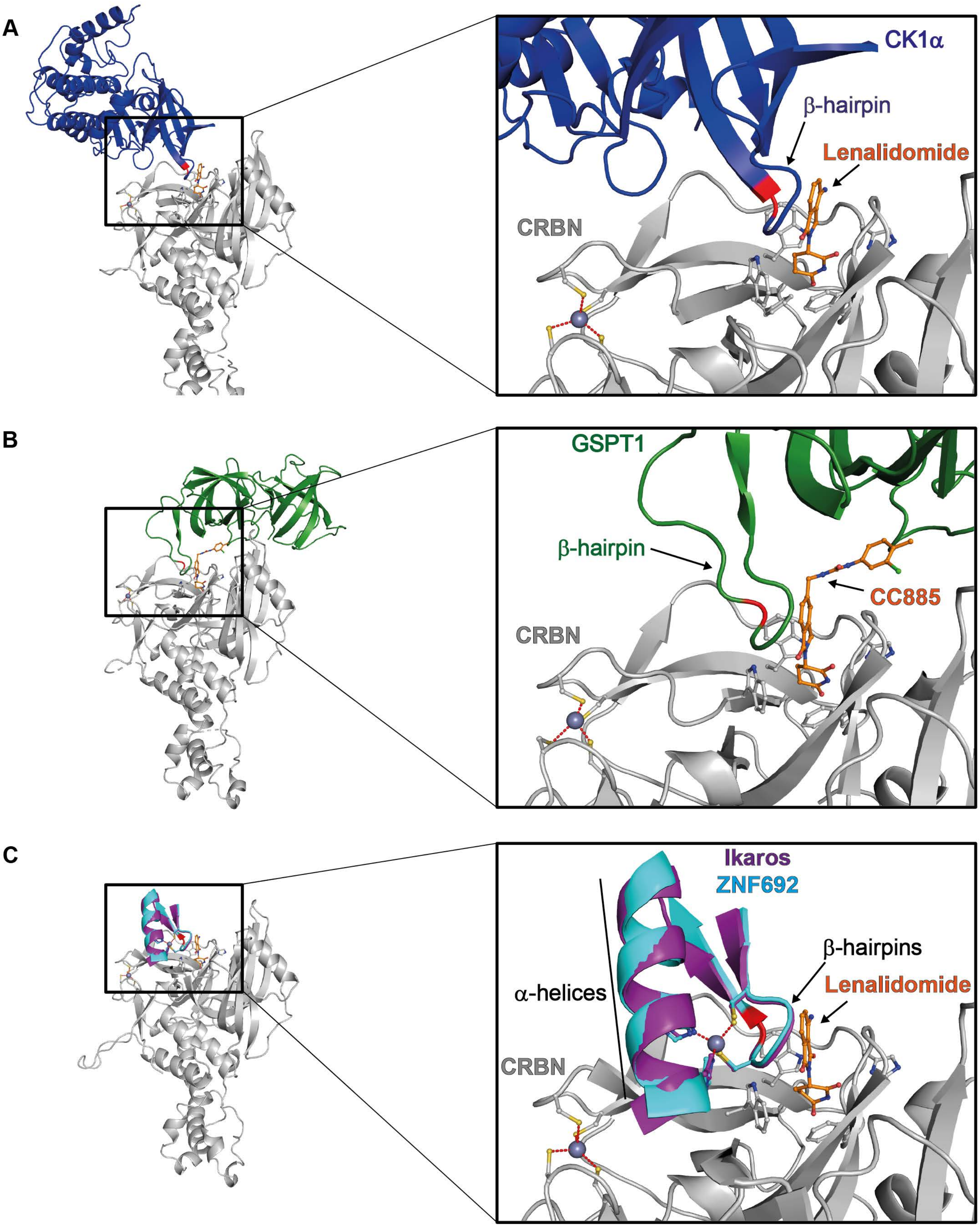

Figure S2. Binding mode of various known classes of neo-substrates on CRBN, related to Figure1
(A) Complex formed between human CRBN, Lenalidomide and human CK1α (PDB 5FQD, Petzold *et al.*, 2016).
(B) Complex formed between human CRBN, CC885 and human GSPT1 (PDB 5HXB, Matyskiela *et al.*, 2016).
(C) Overlay of the complexes formed between human CRBN, Lenalidomide and human Ikaros or ZNF692 (PDBs 6H0F and 6H0G, respectively, Seivers *et al.*, 2018), highlighting the conserved binding mode of both zinc-finger proteins. The crucial Gly of the G-loop degron is highlighted in red.

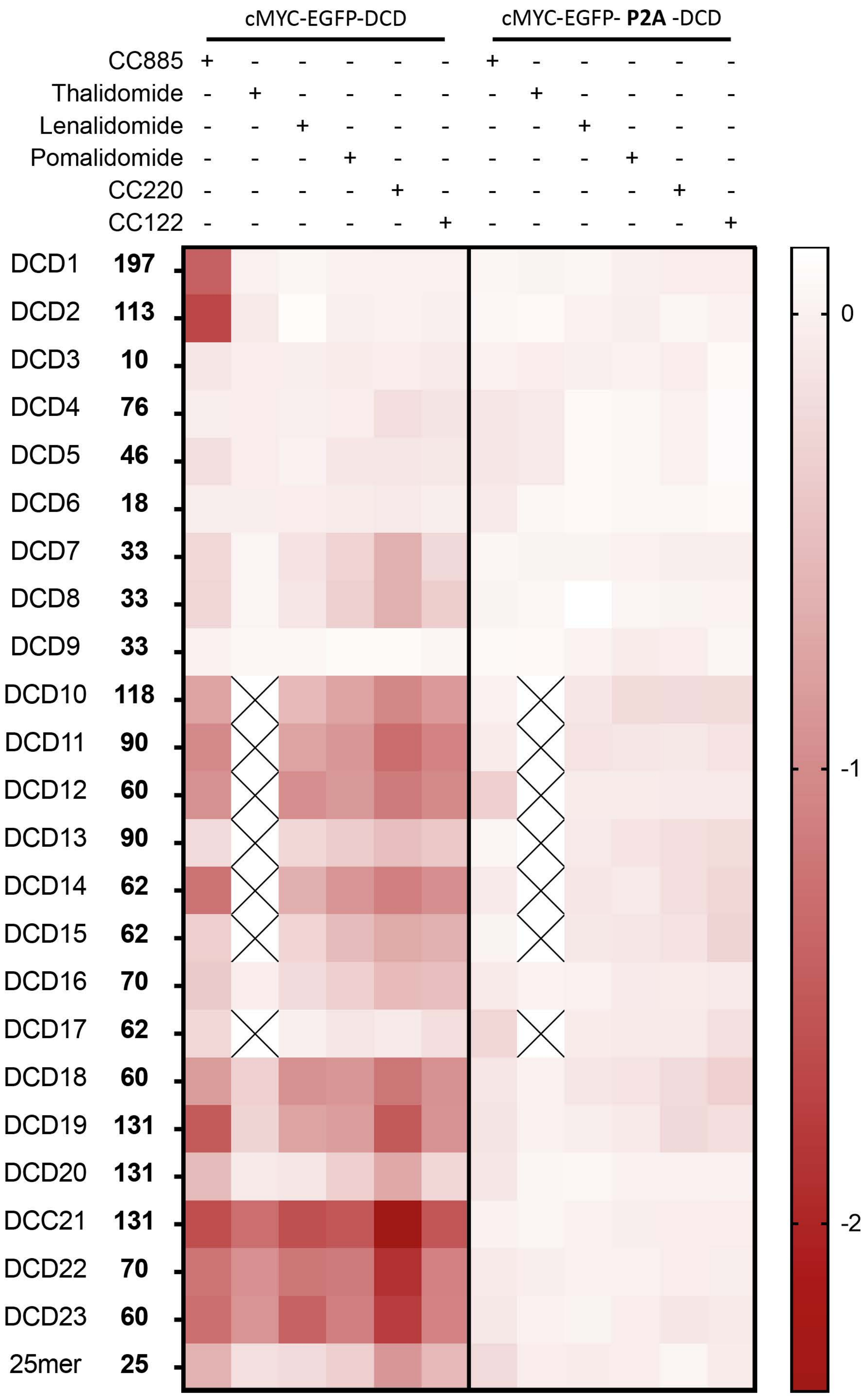

Figure S3. Heat map summary of the degradation matrix data of all the DCDs tested in flow cytometry experiments, related to Figure1

Normalized values were represented in the Log2 Fold Change relative to the DMSO control. In the third group of chimeric ZF DCDs (21-23), DCD21 led to the most potent degradation but had a significantly larger size compared to DCD23 (131 residues vs 60 residues respectively).

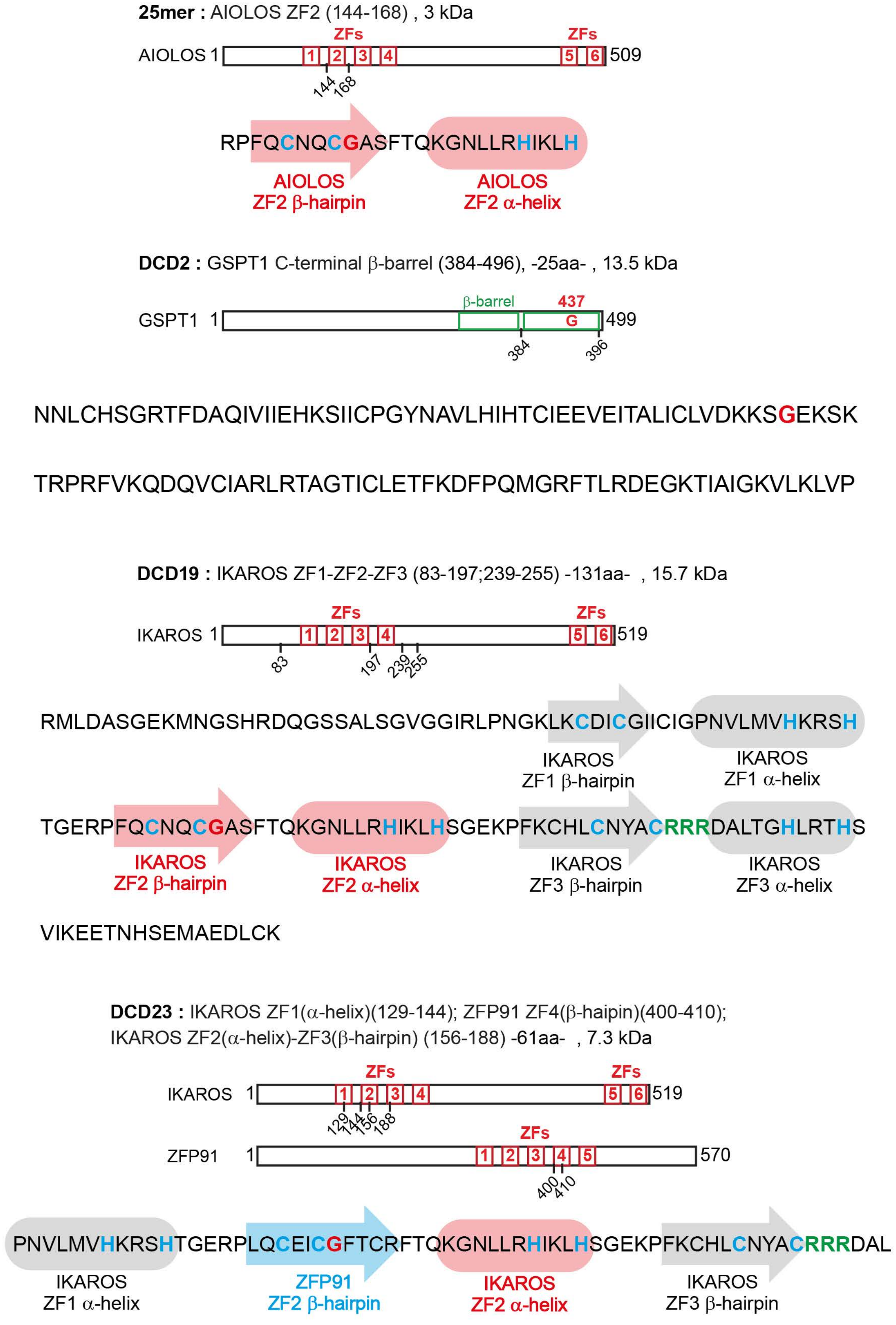

Figure S4. Sequence of Aiolos 25-mer, DCD2, DCD19, DCD23 showing the different critical motifs motifs, related to Figure1

The 2 Cys and 2 His from the C2H2 zinc finger motif are highlighted in blue. The crucial Glycine in the β-hairpin loop degron is highlighted in red. The three arginines in Ikaros ZF3 that were shown to enhance the binding to CRBN are highlighted in green.

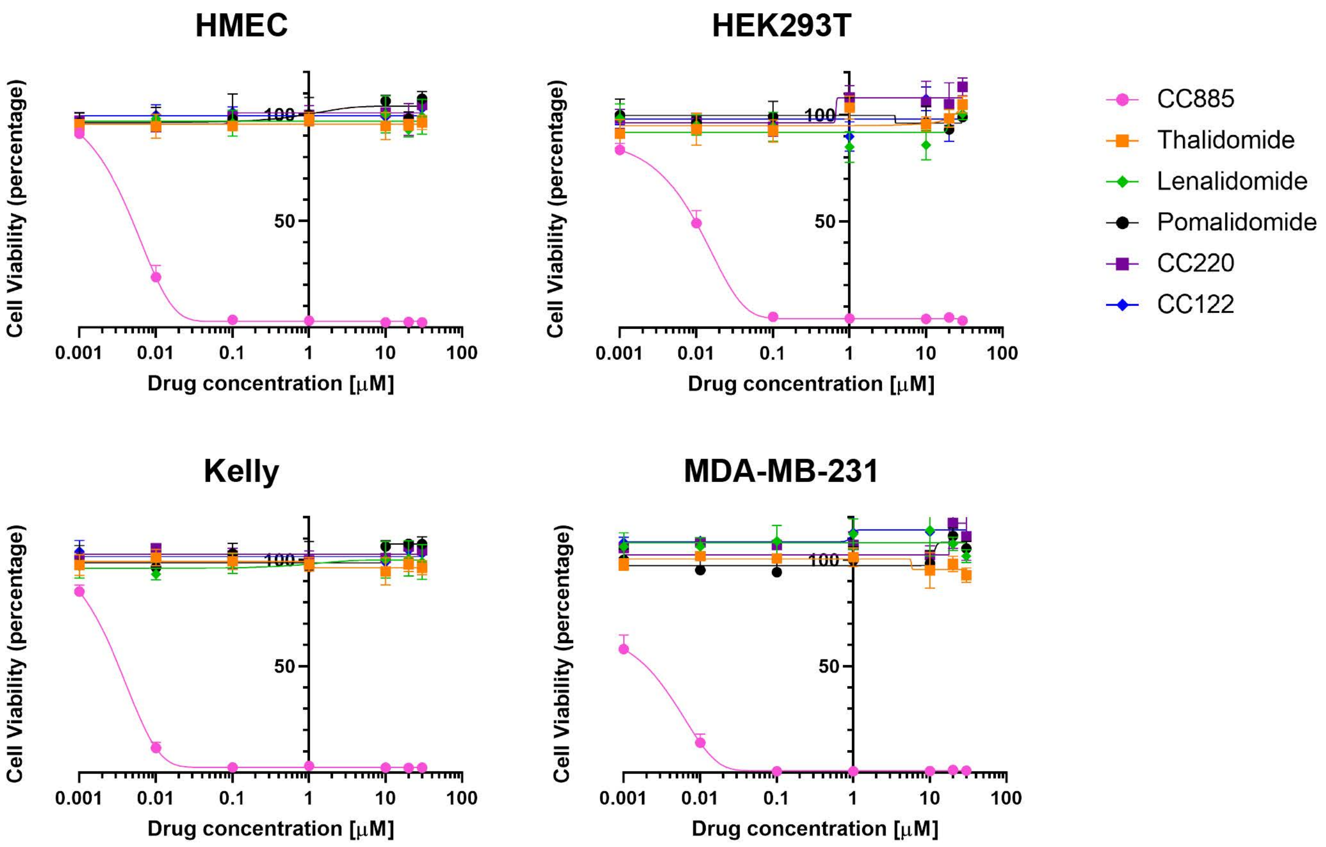

Figure S5. Measure of cellular toxicity of the different degraders used in this study, related to Figure1
Cells were treated with the indicated compounds at different concentrations ranging from 0.001 to 30μM. Cell viability was evaluated using the Promega CellTiter-Blue® Cell Viability Assay. CC885 caused signification cytotoxicity in all the cellular models tested.

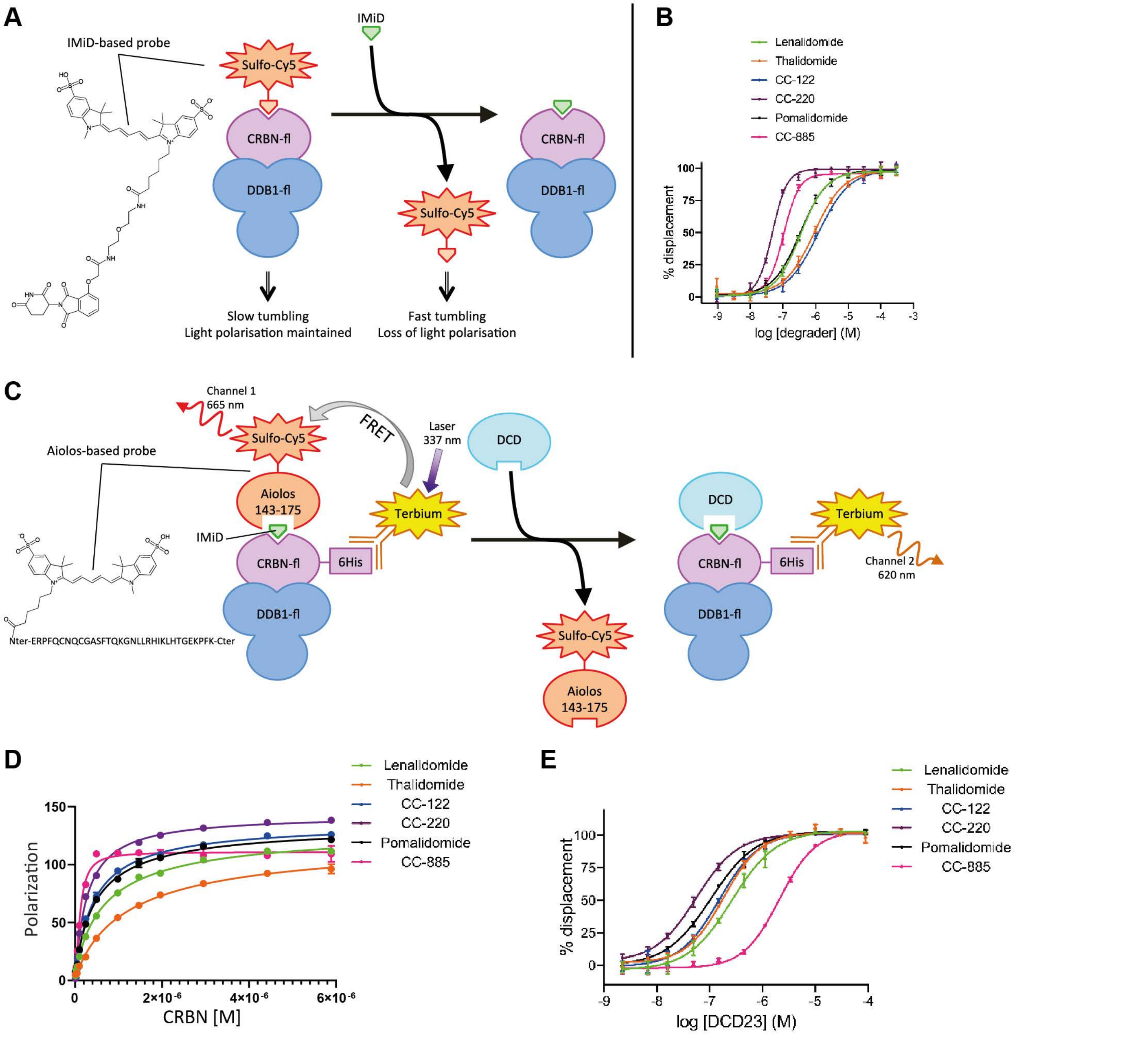

Figure S6. In vitro evaluation of DCD23 binding to CRBN/DDB1 complex, related to Figure1

(A) Schematic representation of the IMiD-based FP assay used to evaluate the relative affinities of the various IMiDs used in this study towards CRBN/DDB1 complex.

(B) IMiD-based FP assay curves obtained for the various IMiDs used in this study. FP IC_{50} in **Table S2.**

(C) Schematic representation of the Aiolos peptide-based TR-FRET assay used to evaluate the ability of IMiDs to promote the binding of DCDs to CRBN/DDB1 complex.

(D) Aiolos peptide-based binding curves in presence of the different IMiDs used in this study. This data was used to define the Kd values in **Table S2.**

(E) Aiolos peptide-based TR-FRET assay curves obtained with DCD23 and the various IMiDs used in this study. IC_{50} and Ki values in **Table S2.**

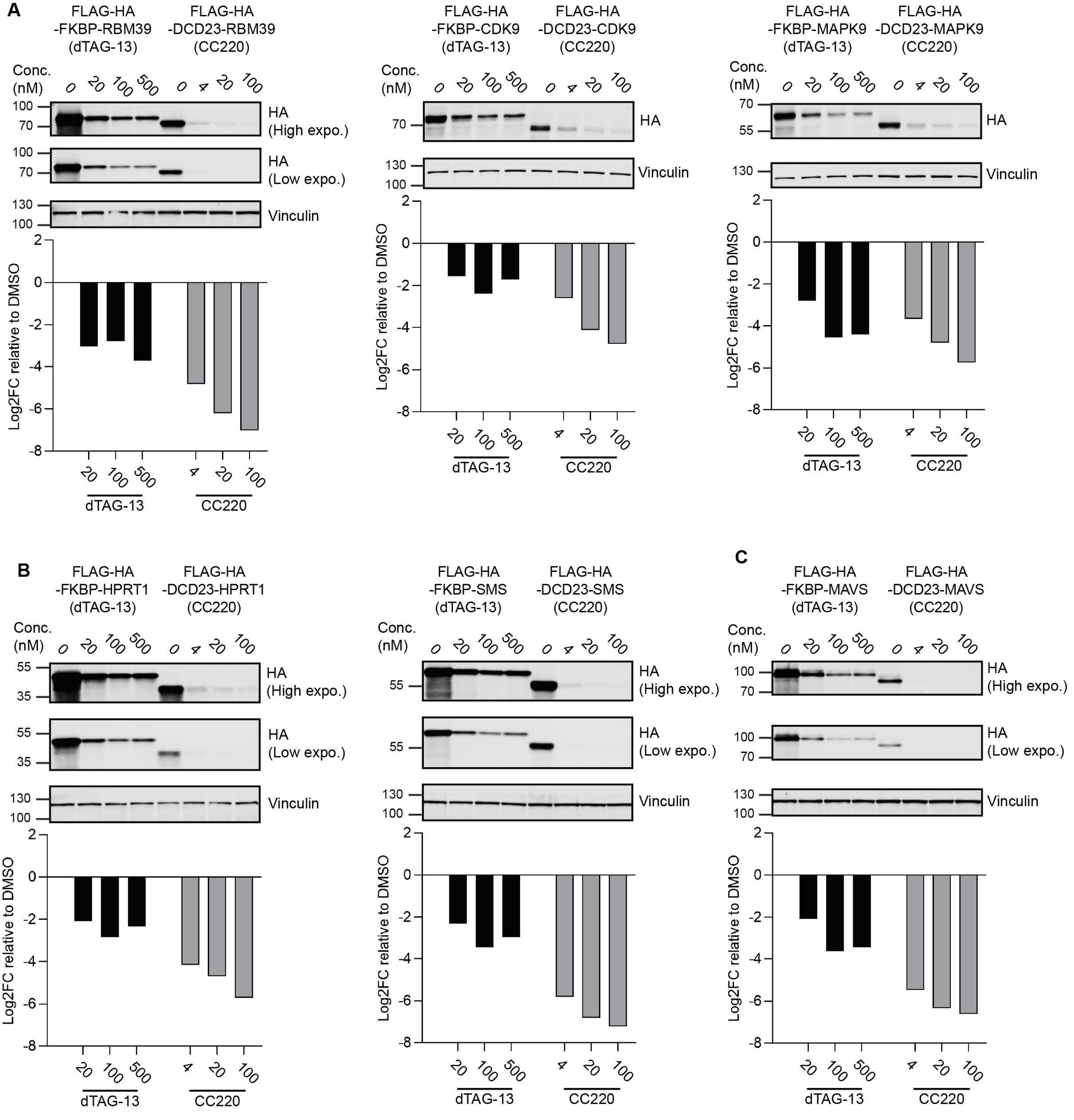

Figure S7. Degradation of targets in various cellular compartments, related to Figure 3

(A-C) SUM159 cells stably expressing various nuclear **A**, cytoplasmic **B** or mitochondrial **C** targets N-terminally tagged with dTAG (Flag-HA-FKBP12[F36V]) or iTAG (Flag-HA-DCD23) were treated with dTAG-13 or CC220 respectively for 24h at the indicated doses. The protein lysates were probed for HA using western blotting. Bands intensities were quantified and represented as Log2 fold change relative to DMSO.

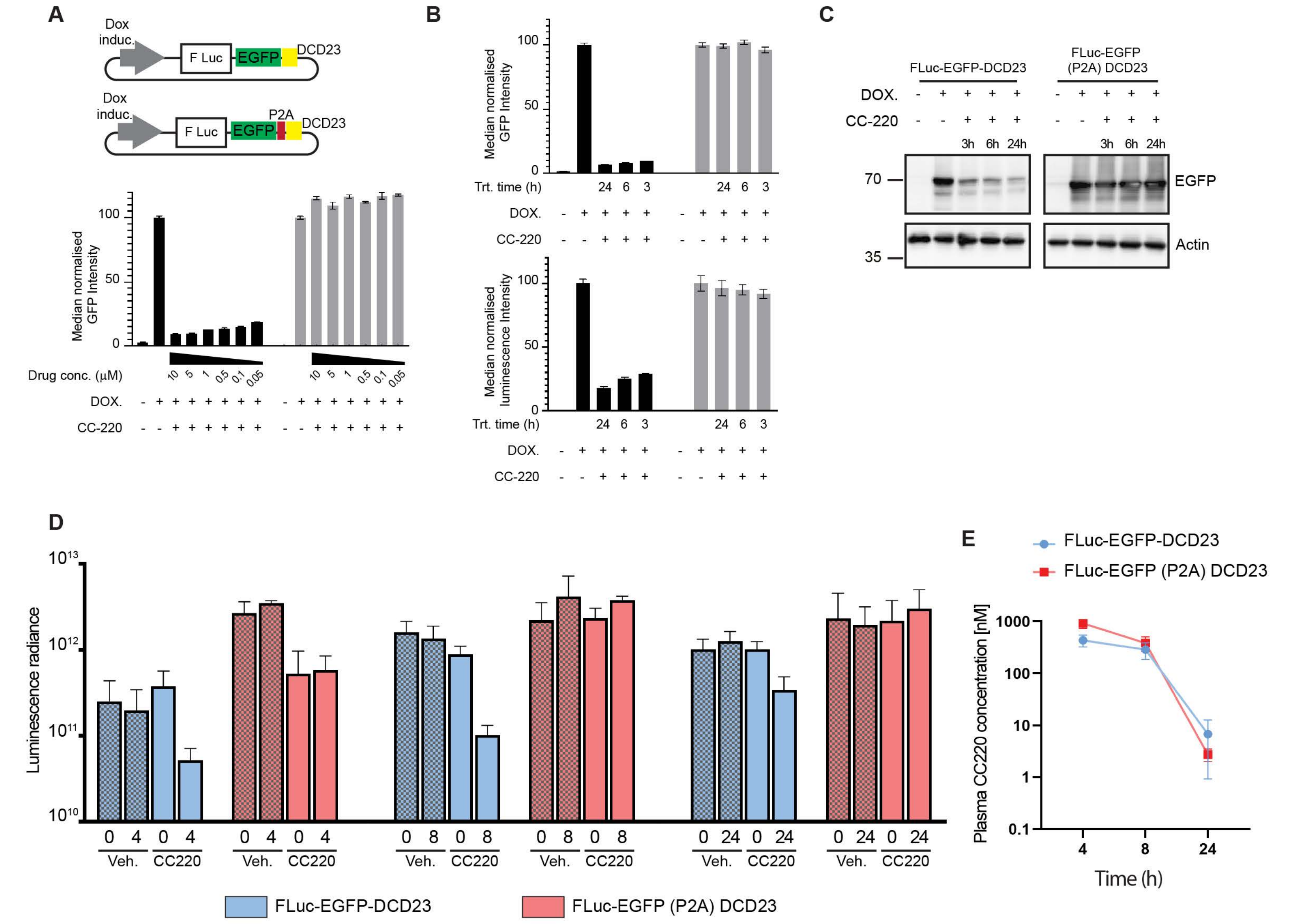

Figure S8. Validation of cellular models for *in vivo* experiment, related to Figure 4

(A) MDA-MB-231 cells stably expressing Dox inducible FLuc-EGFP-DCD23 or the corresponding P2A control were induced with doxycycline for 24h then treated with either DMSO or CC220 for 24h at the different indicated concentration. Median EGFP intensity was measured in each condition using flow cytometry.

(B,C) Time course of Fluc-EGFP-DCD23 loss either by measuring EGFP median intensity, luminescence or Western Blotting **C**.

(D) Quantification of bioluminescence signals from mice represented in **(figure 4A)** and their corresponding vehicle treated mice.

(E) CC220 plasma concentration measured by liquid chromatography tandem mass spectrometry in treated animals.

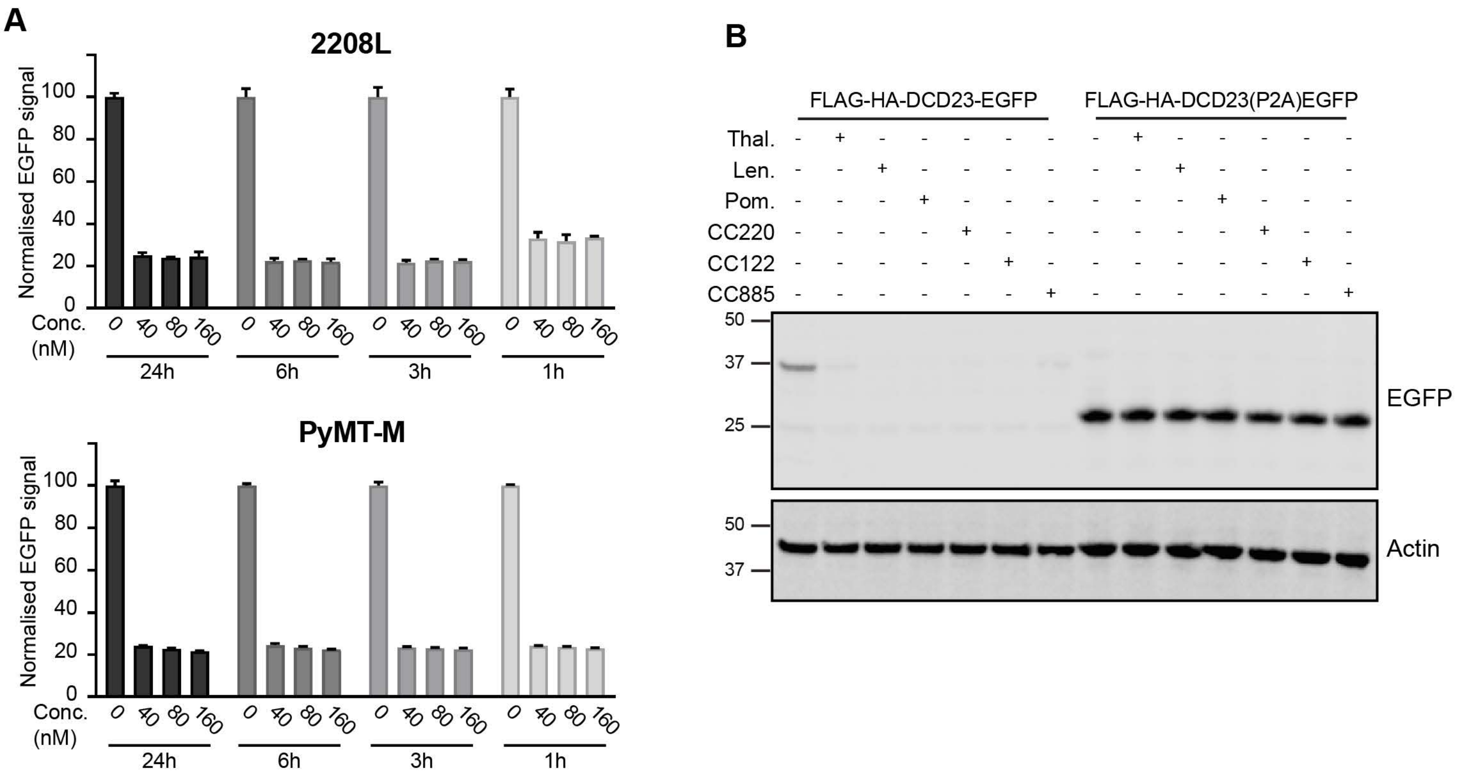

Figure S9. iTAG induced degradation in murine cells, related to Figure 5

(A) 2208L or PyMT-M cells stably expressing Flag-HA-DCD23-EGFP were treated with CC220 for 24, 6, 3 or 1h at the indicated doses. EGFP signal was then measured using flow cytometry.

(B) CT26 cells stably expressing Flag-HA-DCD23-EGFP or the corresponding P2A control were treated with the indicated drug for 24h at 10μM. The protein lysates were probed for EGFP using western blotting.

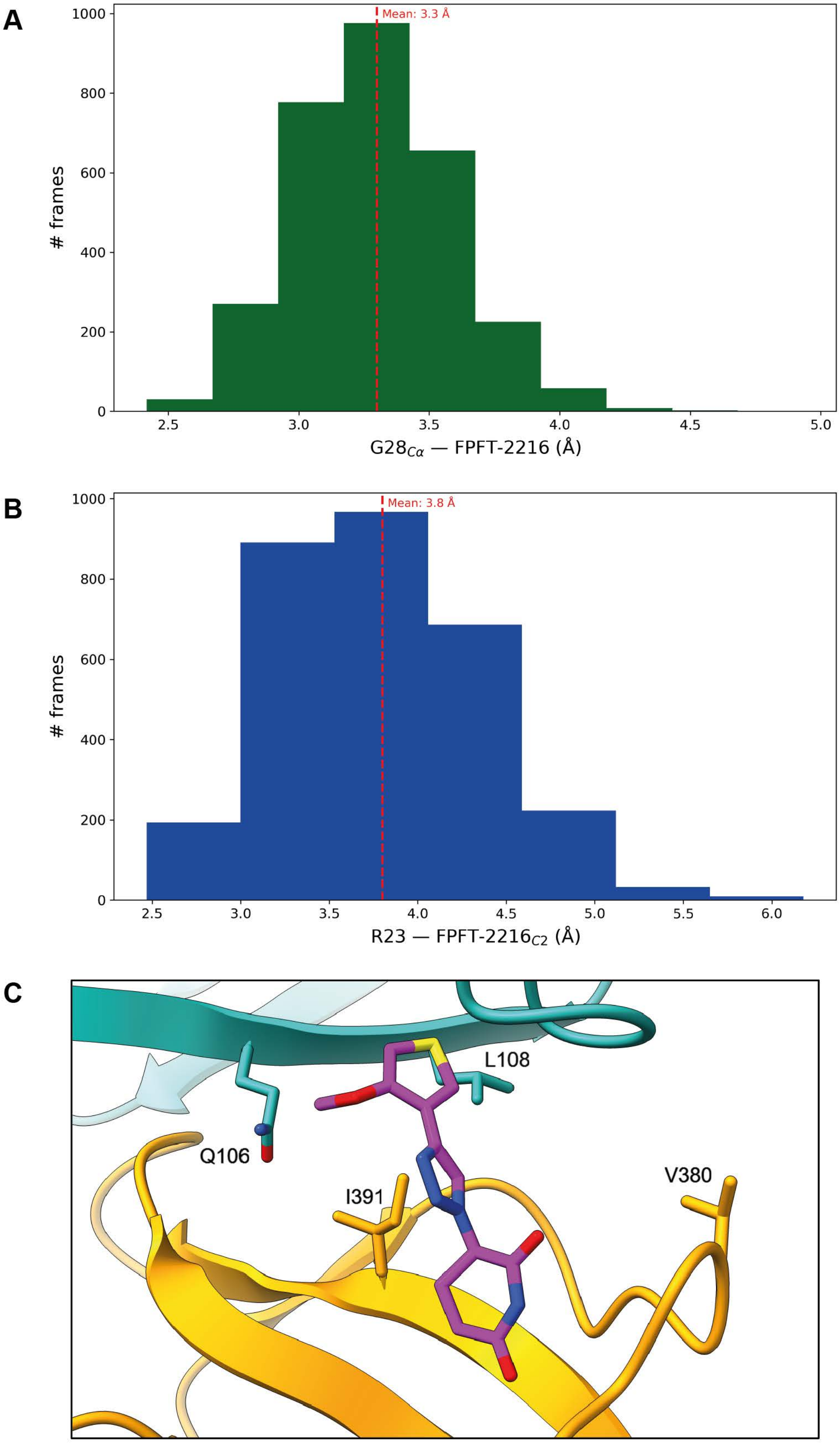

Figure S10. Distance distribution across molecular dynamics trajectory frames, related to Figure 6

(A) Distance between Gly28-Cα of mouse PDE6D and the closest atom of FPFT-2216 in each frame

(B) Distance between Arg23 of mouse PDE6D and the carbon atom adjacent to the methoxy group on the thiophene ring of FPFT-2216.

(C) Mouse PDE6D residues found within 4 Å from Ile391 of mouse CRBN (Val388 in human CRBN).

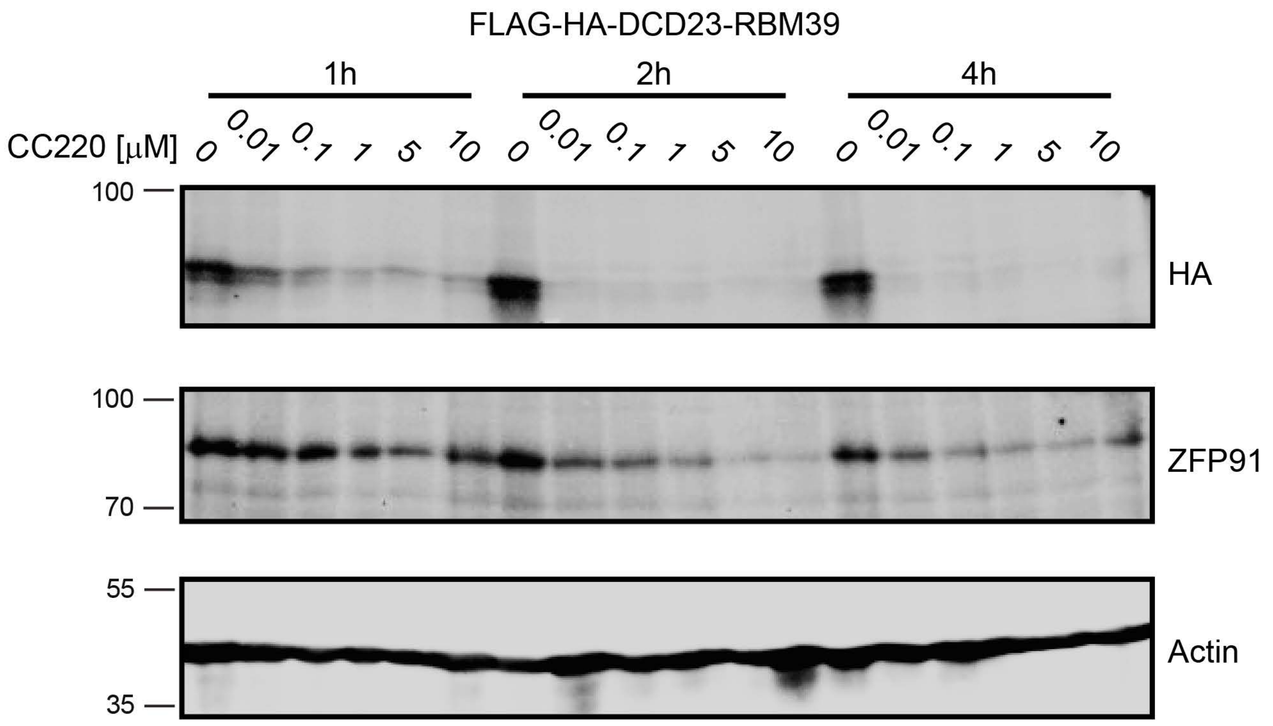

Figure S11. Pleiotropic activity of CC220, related to Discussion
SUM159 cells expressing Flag-HA-DCD23-RBM39 were treated with CC220 for 1, 2, 4h at the indicated doses. Lysates were analysed using western blotting for the degradation of the ZF containing protein ZFP91.

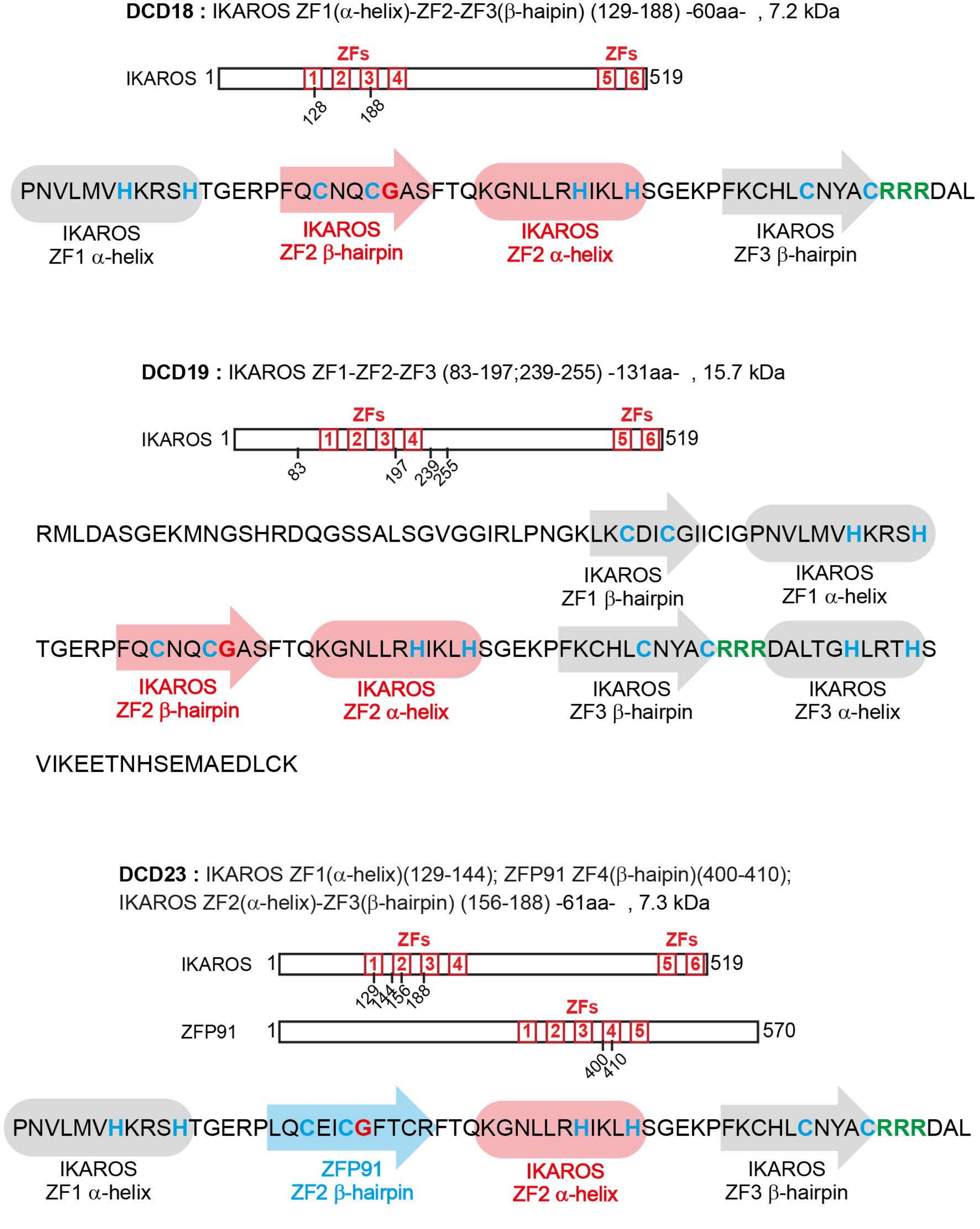

Figure S12: Sequence of DCD18, DCD19, DCD23 showing the different zinc finger motifs, related to Discussion

The 2 Cys and 2 His from the C2H2 zinc finger motif are highlighted in blue. The crucial Gly in G-loop degron is highlighted in red. The three arginines in Ikaros zinc finger 3 that were shown to enhance the binding to CRBN are highlighted in green.

Table S1: Summary of IMiDs/CELMoDs substrate specificity, related to introduction and to Figure1

Data from Thoma and co-workers, Ebert/Fischer and co-workers, Chamberlain and co-workers. Reviewed in (N. Ege *et al* 2021)

Degrader	2D structure	C2H2 zinc finger containing proteins degraded			Non zinc finger containing proteins degraded	
		Ikaros (IKZF1)	Aiolos (IKZF3)	ZFP91	GSPT1	Casein Kinase 1α
**CC885**	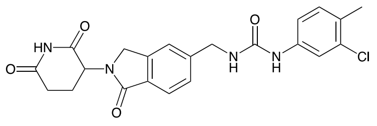	**X**	**X**		**X**	
**Thalidomide**	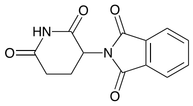	**X**	**X**			
**Lenalidomide**	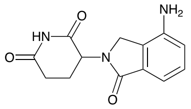	**X**	**X**			**X**
**Pomalidomide**	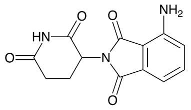	**X**	**X**	**X**		
**Iberdomide/ CC220**	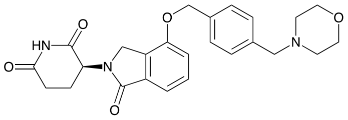	**X**	**X**	**X**		
**Avadomide/ CC122**	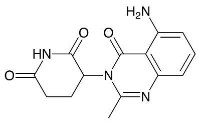	**X**	**X**	**X**		

Table S2: Summary of the *in vitro* binding and EGFP cellular degradation data for the DCD23, related to Figure1

Degrader	2D structure	FP IC_{50} (µM)	Kd (µM)	TR-FRET IC_{50} (µM)	Ki (µM)	Degradation as Log2 FC relative to DMSO
**CC885**	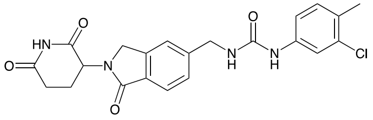	0.107	0.11	2.087	0.266	-1.27
**Thalidomide**	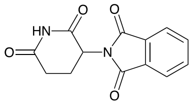	0.911	1.27	0.184	0.115	-0.90
**Lenalidomide**	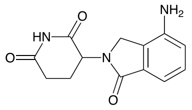	0.353	0.8	0.265	0.136	-1.38
**Pomalidomide**	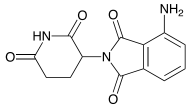	0.342	0.46	0.107	0.040	-1.19
**CC220**	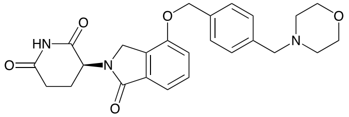	0.048	0.27	0.057	0.015	-1.87
**CC122**	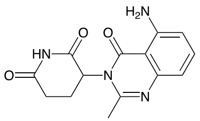	1.208	0.43	0.153	0.055	-1.15
